# Flight in the dark: different responses to darkness in flying insects

**DOI:** 10.1242/jeb.251675

**Published:** 2026-05-20

**Authors:** Roni Maya, Noam Lerner, Bar Karov, Noam Tsory, Richard C. Terrile, Orit Peleg, Tsevi Beatus

**Affiliations:** ^1^ School of Computer Science and Engineering, The Hebrew University of Jerusalem, 9190401 Jerusalem, Israel; ^2^ Department of Ecology, Evolution and Behavior, The Institute of Life Sciences, The Hebrew University of Jerusalem, 9190401 Jerusalem, Israel; ^3^ Center for Bioengineering, The Hebrew University of Jerusalem, 9190401 Jerusalem, Israel; ^4^ Department of Computer Science, University of Colorado Boulder, Boulder, CO 80309, USA; ^5^ BioFrontiers Institute, Departments of Physics, Applied Mathematics, Ecology and Evolutionary Biology, University of Colorado Boulder, Boulder, CO 80303, USA; ^6^ Santa Fe Institute, Santa Fe, NM 87501, USA

**Keywords:** Insect flight, Flight kinematics, Fruit fly, Mosquito, Honey bee, *Culex*, *Drosophila*, *Apis*, Antenna, Low light, Darkness, Perturbation

## Abstract

Vision-based stabilization is crucial for flying animals, and among these, flying insects provide a range of experimental systems for studying vision-related locomotion resilience and sensory integration; for example, under low-light conditions. Behavioral experiments on flying insects can provide a top-down approach for setting limits to their sensory capabilities in such conditions, yet the flight kinematics of insects with minimal visual cues has received little attention. Here, we compared the flight of fruit flies, mosquitoes and honey bees under continuous low light and upon sudden mid-flight darkness. While honey bees typically do not fly in the dark and upon a sudden mid-flight darkness crash within 100 ms, fruit flies and mosquitoes can fly under continuous low-light levels and upon sudden darkness exhibit a well-defined response maneuver. The sequence consists of a visual-motor delay, increasing wingbeat frequency, pitching-up, slowing down and changing course, all within a 250 ms observation time. A similar response was also observed under a sudden transition to partial darkness, which better mimics naturally occurring conditions. The stark differences between the honey bees' and dipterans' responses can be attributed to the dipterans' higher light sensitivity and to the fact that they have halteres – gyroscopic organs that enable them to measure their body attitude regardless of vision. Such free-flight experiments could be combined with further electrophysiological characterization of insect light sensitivity and adaptation to better understand the dynamic role of vision and sensory fusion in flight.

## Teaser

**Summary:** Upon sudden darkness in mid-air, fruit flies and mosquitoes maintain flight and perform a new startle maneuver; in contrast, honey bees crash down.

## INTRODUCTION

For flying animals, the importance of vision-based stabilization is crucial, as low-light conditions or sudden loss of visual signals might impede flight performance and even result in a detrimental crash (Faust, 1952; Stein, 1993; Borst and Groschner, 2023; Dickinson et al., 2000; Taylor and Krapp, 2007; Bar et al., 2015; Souman et al., 2009; Anwar et al., 2024). Flying insects provide a range of experimentally accessible systems, such as diurnal and nocturnal species that have adapted to fly in different light conditions (Faust, 1952; Sponberg et al., 2015; Reber et al., 2015; Müller and Robert, 2001; Baird et al., 2015; Leonte et al., 2021; Goulard et al., 2016; Verbe et al., 2023), for studying vision-related locomotion resilience and sensory mechanisms (Borst and Groschner, 2023; Taylor and Krapp, 2007; Sane, 2003; Dickinson and Muijres, 2016; Sun, 2014; Wessnitzer and Webb, 2006; Stewart et al., 2010; van Breugel et al., 2015; Vinauger et al., 2019). Although much is known about the visual system of many insects, understanding what useful visual information they extract under low-light conditions is far from trivial (Borst and Groschner, 2023; Juusola and Song, 2017). For example, the insect compound eyes are generally categorized into two types, apposition and superposition eyes (Warrant, 2017). In apposition eyes, each ommatidium is optically coupled to one distinct set of photoreceptors. This architecture favors spatial resolution over light sensitivity and is, hence, more common in diurnal insects such as honey bees. In superposition eyes, a single photoreceptor collects light from a number of facet lenses, which favors light sensitivity over spatial resolution. Yet, this distinction is not clear cut, as there are nocturnal insects that see remarkably well in the dark despite having apposition eyes (Warrant, 2008, 2017; Frederiksen and Warrant, 2008; Honkanen et al., 2014; Reid et al., 2011), such as sweat bees (*Megalopta genalis*) that can fly in extremely low light conditions (Warrant et al., 2004), and mosquitoes that have apposition eyes but with broad rhabdoms which may increase their light sensitivity (Land et al., 1997). In contrast, fruit flies, which are not nocturnal (Palavalli-Nettimi and Theobald, 2020), have superposition eyes (Warrant, 2017). Apart from the sensitivity at the photoreceptor level, which was measured in dark-adapted insects to be as high as enabling single photon detection (Honkanen et al., 2014; Henderson et al., 2000; Hardie and Juusola, 2015), it has been recently shown that in fruit flies, low-light adaptation is a complex process that takes place both at the photoceptor level and at the downstream neural level (Ketkar et al., 2023). The duration of this combined adaptation process, measured from 160 to 0.04 lx is of the order of 1 s. In honey bees (*Apis mellifera*), full light adaptation at the photoceptor level takes 10–20 min (Wolf and Zerrahn-Wolf, 1935; Kindermann and Hertel, 1986).

Behavioral experiments on flying insects provide a complementary, top-down approach for setting limits to the capabilities of their sensory mechanisms and motion control under low-light conditions or following the sudden reduction of visual cues. For example, field studies have shown that although honey bees stop foraging at light levels of 50 lx and that their outdoor activity practically stops at 10 lx (Kevan and Baker, 1983), the nocturnal sweat bees (*M. genalis*) can fly and navigate by landmarks under starlight only, at extremely low light intensity of 10^–4^ to 10^–5^ lx (Baird et al., 2015; Warrant et al., 2004; Theobald et al., 2007), though with reduced landing performance (Theobald et al., 2007; Kelber et al., 2006). Light intensity also sets the limit of foraging flight initiation in the crepuscular *Lasioglossum* (*Sphecodogastra*) *lusoria* bees, with no flights under 0.06 lx illuminance (Kelber et al., 2006), and *Vespa crabro* hornets in a flight-mill assay were shown to fly slower under 0.5 lx than under 850 conditions (Spiewok and Schmolz, 2006). In flower-tracking hawkmoths, Sponberg et al. (2015) compared tracking trajectories at 300 and 0.3 lx (the latter is equivalent to moonlight), showing that tracking performance was degraded as a result of longer visual processing delay (Warrant, 2015). Tethered-flight experiments by Palavalli-Nettimi and Theobald (2020) have shown that fruit flies can detect and respond to a striped pattern at light levels as low as 0.3 lx but as light levels decrease towards this level, the flies gradually lose spatial, contrast and temporal acuity. Reber et al. (2015) conducted both free-flight experiments and electrophysiological measurements of the photoreceptors in *Bombus* at light intensities as low as 3.4 lx. This work further established a tradeoff of flight at low-light levels: photoreceptor adaptation to low light includes an increase in its integration time, which comes at the cost of decreasing both the spatial and temporal visual resolutions. Because of the resolution loss, insects slow down their flight speed, presumably to reduce motion blur, and their flight trajectories became more tortuous. Currea et al. (2022) demonstrated this tradeoff between spatial and temporal acuity when comparing fruit flies (*Drosophila melanogaster*) and desert flies (*Drosophila mojavenesis*) both morphologically and using tethered-flight assays under different light levels down to 3 lx.

Another body of work characterized the flight performance of various insect species under dark conditions. In 1952, Faust tested insects of several orders in free flight, characterizing whether they continue flying after the ambient light is turned off (Faust, 1952). These observations, which were analyzed based on the insects’ buzzing and their sound crashing to the ground, showed that the responses differed significantly between orders. For example, while Hymenoptera and Heteroptera stopped flying and crashed immediately after the light turned off, flies and mosquitoes (Diptera) kept flying in the dark. Müller and Robert (2001) characterized the phonotactic flight trajectories of the parasitoid fly *Ormia ochracea*. Females of this species, which lay their eggs on calling male crickets, were able to fly in the dark and locate a target speaker transmitting these calls. Goulard et al. (2016, 2018) tested *Episyrphus balteatus* hoverflies that were dropped upside-down in different visual arenas and light conditions. When flying in a lit environment with visual features, the hoverflies managed to turn over and recover their attitude. Yet, they were unable to recover and crashed significantly more frequently under several conditions: in the dark, when the light came from the bottom and when the visual arena was lit but featureless. It was concluded that at the measured time scale of ∼300 ms, hoverflies estimate where down is mostly using their visual system, by assuming light comes from above. In a subsequent experiment, Verbe et al. (2023) showed that leg proprioceptive information prior to the upside-down drop was sufficient for the hoverflies to properly align their roll angle, under most of the tested conditions. In fruit flies, flight in the dark was measured by Leonte et al. (2021) in the context of demonstrating the importance of motion vision in free flight. First, they reported that fruit flies are reluctant to take off in the dark, and had to be forced to do so using a vibratory device. Second, by measuring the flies’ center-of-mass trajectories in a 50×50×30 cm arena, they observed that fruit flies can fly in the dark and do so faster and less straight than in lit conditions. Although these studies did not specify the light intensity used in the dark conditions, they provide a valuable comparison of the flight performance of many species.

The above studies raise several questions, such as: what are the flight kinematics of insects upon sudden darkness; and are there differences between the kinematics of different fliers under such conditions? Here, we compared the responses of *D. melanogaster* fruit flies, *Culex pipiens* mosquitoes and *A. mellifera* honey bees to either constant or sudden darkness during free flight. These three species represent markedly different lifestyles: *D. melanogaster* is a solitary crepuscular/diurnal forager (Palavalli-Nettimi and Theobald, 2020) that mates and lays eggs on surfaces, *C. pipiens* is a solitary nocturnal parasite that mates during flight and lays its eggs on water surfaces, and *A. mellifera* workers are social diurnal foragers. Moreover, their wing kinematics and flight-related sensory mechanisms are different, where the prime difference is that Diptera have halteres – gyroscopic organs that measure the insect's angular velocity based on the Coriolis effect (Pringle, 1948; Nalbach, 1993; Yarger and Fox, 2016; Dickerson et al., 2019) – whereas bees do not have such sensory organs. We show that while honey bees do not fly in the dark and immediately stop flying and crash upon the onset of darkness in mid-air, fruit flies and mosquitoes can fly in the dark and, in addition, upon sudden darkness in mid-air they resume stable and controlled flight, at least within our 250 ms observation time. Moreover, despite their different flight kinematics, visual systems and lifestyles, both dipterans exhibit a similar and previously unreported response to sudden darkness with a well-defined sequence: after a visual-motor delay of ∼45 ms, they increase their wingbeat frequency, pitch up to brake their velocity, and change course. A similar response of these two species was also observed under a transition to partial darkness, which better mimics naturally occurring conditions. This response is qualitatively different from the directional looming responses in fruit flies (Muijres et al., 2014) and mosquitoes (Cribellier et al., 2022, 2024). Our findings enable this darkness response to be further used for studying sensory and neural mechanisms in biolocomotion.

## MATERIALS AND METHODS

### Animals

We used female *Drosophila melanogaster* Meigen 1830 fruit flies (Canton-S line), 3–7 days old, grown at 25°C and 70% humidity, on standard fly food and under a 12 h:12 h light:dark cycle. We used male *Culex pipiens* Linnaeus 1758 mosquitoes obtained from a stable laboratory colony. Male mosquitoes were selected and fed with sugar–water under a 12 h:12 h light:dark cycle, at 70% humidity and 25°C. We used *Apis mellifera* Linnaeus 1758 honey bees grown in an outdoor standard commercial hive.

### Experimental setup

**Fig. 1.**
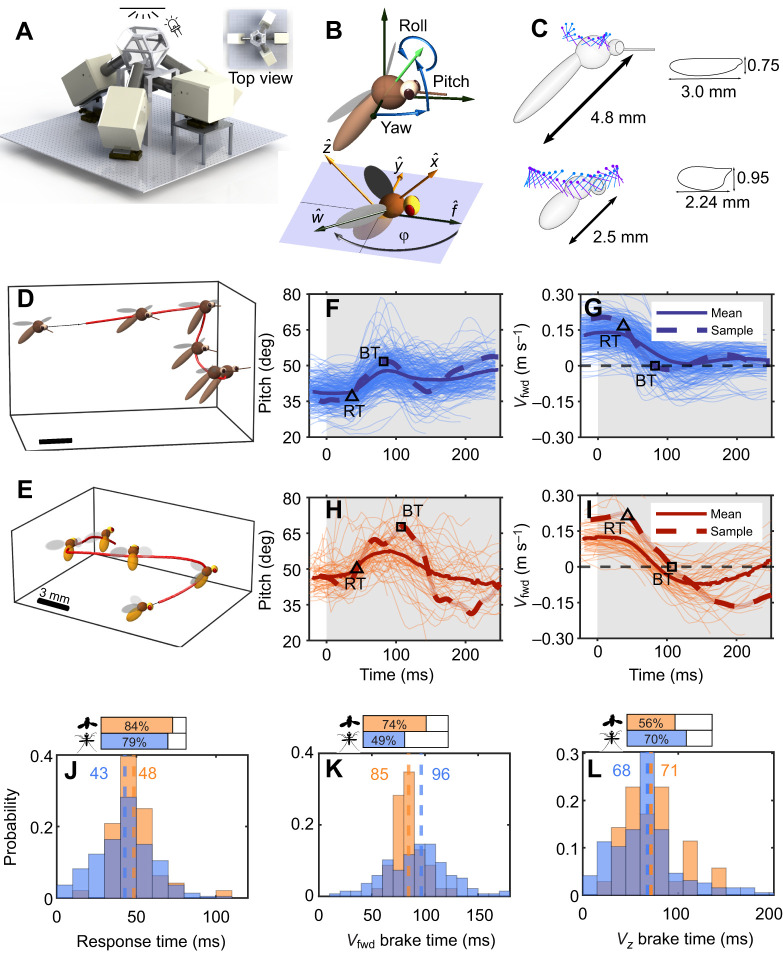
**Fruit flies and mosquitoes exhibit a well-defined response upon a darkness ‘step’ perturbation.** (A) The experimental setup consisting of four fast cameras and a flight chamber. The position of the visible LED light source is indicated (see Materials and Methods). (B) The insect's body and wing axes and degrees of freedom. (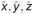), body axes; 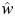, wing axis; (yaw,pitch,roll), body Euler angles; φ, stroke angle; 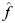, forward-facing axis (the projection of the body *
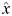
*-axis onto the *xy* plane). (C) Typical body and wing dimensions, and wing kinematics for both species. Blue, forward stroke; purple, back stroke. (D,E) Representative 3D trajectories of a mosquito (D) and a fly (E); trajectories and insects are plotted to scale and marked red during darkness. (F–I) The entire dataset of step perturbation, with means (solid bold lines) and representative events (dashed lines). Pitch angle and forward velocity for mosquitoes (F,G) and flies (H,I). RT, response time; BT, brake time (*V*_fwd_=0) are marked for the representative events. (J–L) Distributions of response times: (J) *V*_fwd_ brake times (K) and *V_z_* brake times (L) for the mosquitoes (blue) and flies (orange). Means are marked in the same colors. Insets show the percentage of events that meet each criterion.

Fruit flies or mosquitoes were released in a transparent flight chamber and filmed by four synchronized and calibrated (Theriault et al., 2014) fast cameras (Phantom v2012, Vision Research; Fig. 1A). The cameras were backlit by infrared LEDs at wavelength of 810 nm (Osram, SFH-4780S), which neither species can detect (Sharkey et al., 2020; Gibson, 1995; Yamaguchi et al., 2010; Muir et al., 1992). The joint filming volume of the cameras was equivalent to a cube of side length 3.5 cm, located at the center of the flight chamber, 10 cm from its floor and sidewalls. Imaging resolution was 1280×800 pixels. The frame rates used were 16,000 frames s^−1^ for the fruit flies (∼70 frames per wingbeat), and 20,000 frames s^−1^ for the mosquitoes (∼31 frames per wingbeat). Further details are given in Maya et al. (2023).

To control the light conditions, the entire setup was covered by an opaque cloth and an upward-pointing white LED (Osram, LW-G6SP) was placed outside the flight chamber and inside the cloth-enclosed volume to provide controlled ambient lighting that was scattered off the inner white surface of the enclosure. The white LED current was controlled using a constant-current power supply. Visible light intensity was measured using a light meter (Ophir, PD300-uv) when the infrared back-illumination was off. In the lit condition, light intensity was 5 μW cm^−2^ (34 lx), which is similar to typical living-room lighting. In the dark condition, with both white and infrared LEDs off, light intensity was 10 pW cm^−2^ (6.8×10^−5^ lx), which is lower by a factor of 500,000 than in the lit condition and is equivalent to half the light intensity of an overcast night with no moonlight or starlight. In the partial darkness experiments, light intensity was controlled by setting the current on the white LED to obtain a 10-fold reduction in light intensity with respect to the fully lit condition, i.e. 0.5 μW cm^−2^ (3.4 lx). The ambient lighting and video recording were controlled via an Arduino microcontroller. When an insect entered the filming volume, a trigger signal generated by the cameras’ image-based auto-trigger was used to initiate camera recording as well as a sudden darkness or partial darkness perturbation either by turning off the white LED for a predetermined duration or by reducing its intensity 10-fold. Full change of the ambient LED light was obtained within tens of nanoseconds. After each event, the video acquired by each camera was automatically saved to a computer.

For honey bees measurements, ∼30 individuals were placed in a mesh chamber of size 40×40×60 cm (width×depth×height), larger than the flight chamber used for fruit flies and mosquitoes as the bees are larger and require more flight space. The room had no windows nor any light source. The bees were recorded using two cameras (Sony FDR-AX53 Handycam), operated at 60 frames s^−1^ in night mode. As the bees did not fly in the dark or exhibit stable flight under darkness perturbation, imaging at this frame rate was sufficient to characterize their response. Visible light illumination was provided by a white ring LED that was positioned above the chamber. Sudden darkness perturbation was induced by manually turning off the visible light source when one or more bees were flying. Visible light illuminance was measured by a light meter (Thor Labs, PM100A with a S130C sensor). Illuminance in the lit condition was 2600 lx and in the dark condition it was 9.6×10^−4^ lx (light:dark ratio of ∼2,700,000). Infrared light for recording in the dark was provided by two commercial flood light projectors at a wavelength of 850 nm, which bees cannot detect (Menzel et al., 1986).

### Experimental procedure: fruit flies and mosquitoes

Camera calibration was performed by tracking the position of a calibration ‘wand’ object and using the EasyWand toolbox for Matlab (Theriault et al., 2014). Following calibration, animals were placed in the flight chamber and the experiment was operated automatically.

For fruit flies, each experiment included 10–30 animals, free to fly in the experimental chamber. Flies were provided with standard food medium inside the chamber. We recorded and analyzed a total of 652 fruit fly events, divided as follows: 75 events of 5 ms dark pulse, 46 events of 10 ms dark pulse, 90 events of 20 ms dark pulse, 59 events of 40 ms dark pulse, 119 events of 60 ms dark pulse, 89 events of 80 ms dark pulse, 57 events of 100 ms dark pulse, 62 ‘step’ perturbation events and 55 events of 100 ms pulse without the aristae. As control, we measured 322 flight events under constant light conditions, and used these data to characterize the flies' nominal flight. The typical duration of the fruit flies flight events was 180–250 ms depending on the flight speed.

For mosquitoes, we performed two experiments: one with intact animals and the other with arista-removed animals. Each experiment included 10–15 animals that were provided with cotton soaked in sugar water for food. The first experiment lasted for 3 days, during which the perturbation duration was changed several times to collect sufficient data. The second experiment lasted for 2 days, where only step perturbation was applied. Overall, 727 events were selected for analysis, divided as follows: 82 control events under constant lighting, 5 events under constant darkness, 280 events of step dark perturbation, 132 events of 60 ms dark pulse, 119 events of 100 ms dark pulse and 109 events with arista-removed mosquitoes under step perturbation. The typical duration of the mosquito flight events was 250 ms. Arista removal in both fruit flies and mosquitoes was performed under a binocular microscope (Zeiss, Stemi 508), using dissection scissors. In each treated animal, both aristae were cut at their base.

Recording of the flight of fruit flies and mosquitoes under constant darkness conditions was performed as described above. Namely, the cameras were triggered when an insect voluntarily took off and entered the filming volume, which is located in the middle of the flight chamber. The insects exhibited stable flight, e.g. straight or ascending, with no signs of instability of impeded controllability. The occurrence of these flight events, in terms of the number of events per unit time, led to the quantification of the 1:15 and 1:6 takeoff ratio compared with flight under full-light conditions (Table 1).

**Table 1.** Nominal free-flight parameters of mosquitoes, fruit flies and honey bees in lit conditions

	Mosquito	Fruit fly	Honey bee
Mean body mass (mg)	0.7	1.1	116
Speed (m s^−1^)	0.19±0.06	0.10±0.04	7.5 (top speed outdoors)
Wingbeat frequency (Hz)	655±42	233±13	227±13
Wing stroke amplitude (deg)	44±3	142±8	87±8
Body pitch angle (deg)	35.7±5.4	44.5±4.7	39.6±3.7
Takeoff ratio in dark:light	1:6	1:15	0:1

The table summarizes the mean parameters over 322 flight events of fruit flies (*Drosophila melanogaster*) and 82 mosquito (*Culex pipiens*) flight events, all under lit conditions (see Materials and Methods). Honey bee (*Apis mellifera*) data were from Vance et al. (2014) and Wenner (1963). Under continuous darkness, fruit flies and mosquitoes took off significantly less often than in lit conditions, while honey bees did not fly at all in the dark (bottom row). Data are means±s.d.

For both fruit flies and mosquitoes, the flight events were selected for analysis based on their duration and visibility in all camera views.

### Experimental procedure: honey bees

Approximately 30 bees were collected from a standard commercial outdoor hive and placed in the experimental chamber. Bees were fed *ad libitum* with honey on a Petri dish placed at the bottom of the chamber. The two cameras were calibrated using the Matlab Camera Calibration Toolbox. The camera LCD screen was covered to keep the room as dark as possible. First, we recorded 1 h of the bees in lit conditions, during which the bees were flying continuously. Second, we recorded the bees for 1 h in the dark. No bee took off during that part of the experiment. Third, we turned the light back on, and manually turned it off when one or more bees were airborne. The light was turned back on a short while after the bees stopped flying, which was evident by the sound of their buzzing stopping, followed by the audible impact of them crashing into the wall or ground.

### Quantification and statistical analysis

We tracked the body and wing kinematics of free-flying *D. melanogaster* using the hull reconstruction–reprojection method (Maya et al., 2023). This open-source MATLAB-based method reconstructs a 3D hull of the fly's body and wings and estimates their kinematics. This method utilizes the reprojection of the reconstructed 3D hull in order to resolve some of the occlusion generated by the limited number of cameras used. In this work, we used the body kinematics – position and orientation versus time – obtained by this method. We then processed the data in Python to apply filtering and smoothing and calculate velocities and accelerations as described below. Outliers in body and wing angles were removed using *Z*-score filtering (window size of 200 frames), where points with a *Z*-score value greater than 3 were discarded. We then smoothed both the body coordinates and wing angles using the Savitzky–Golay filter implemented by the scipy.signal library. Body kinematic data were smoothed with a polynomial order of 3 and a window size of 511 frames, and the wing kinematics data were smoothed using polynomial order of 2 and window size of 15 frames. Wing kinematics were used for calculating wingbeat frequency. Velocity and acceleration were computed as the first- and second-order derivative calculated by the Savitzky–Golay method.

After filtering and smoothing, we obtained the fly's body and wing degrees-of-freedom (DOF): the body center-of-mass position (*x*,*y*,*z*) in the lab frame of reference, the body's three Euler angles yaw φ_b_, pitch θ_b_ and roll ѱ_b_, and the stroke angle φ for each wing (Fig. 1B). For fruit flies, the body pitch angle is defined as the principal axis of the entire body, including the abdomen, thorax and head. For mosquitoes, the body pitch angle is defined as the principal axis of the abdomen and thorax only, because the head and proboscis, which are difficult to separate in 3D, form a different angle with respect to the lab *xy* plane. For fruit flies, to separate individual wingbeats, we used the find_peaks function in the scipy.signal Python library, on the stroke angle φ sequence, with a ‘prominence’ threshold of 20. A wingbeat was defined as the interval between two consecutive peaks of φ. The wingbeat frequency in Hz for each wing-stroke was calculated as frame rate/*N*, where the frame rate was 16,000 Hz and *N* is wingbeat duration in frames. Additionally, for each event, we manually measured the time at which the fly started extending its legs.

For mosquitoes, we tracked the body kinematics using a neural network model for extracting features in 2D images, which is based on the LEAP model (Pereira et al., 2019), implemented in Python. Using the camera calibration matrices, we triangulated the 2D feature points to find their corresponding 3D points. Each of the (*x*,*y*,*z*) coordinates of these points was smoothed in time using a low-pass filter (MATLAB lowpass function with a 50 Hz cutoff frequency) and then used to construct vectors from which the DOF of the body were calculated (Fig. 1B). The wingbeat frequency was calculated directly from the video footage, relying on the fact that the periodicity of the number of pixels of the mosquito is identical to its wingbeat frequency. The frequency was obtained by performing a fast Fourier transform on the sequence of the number of pixels, in windows of 46 ms (corresponding to ∼30 mosquito wingbeats), equivalent to averaging the wingbeat frequency in each window.

**Fig. 2.**
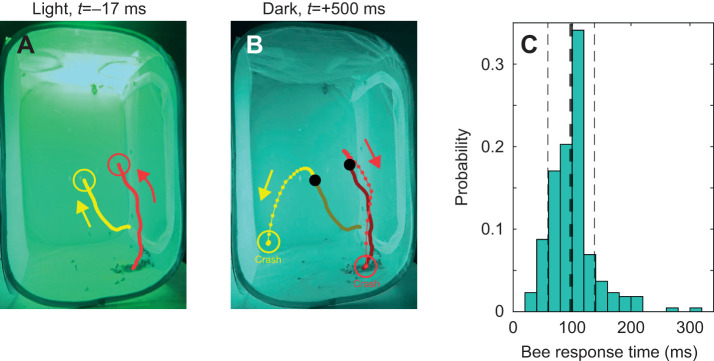
**Honey bees persistently crash ∼100 ms after the onset of darkness.** Approximately 30 bees were recorded in a mesh cage at 60 frames s^−1^. In each event, the overhead white LED light was turned off at *t*=0. In parallel, the chamber was illuminated by infrared flood lights (wavelength of 850 nm) that allowed recording in the dark (see Materials and Methods). (A,B) Representative trajectories of two bees marked in yellow and red. (A) An image taken in the light at *t*=−17 ms, one video frame before the onset of darkness. Bees' positions at that time are marked by circles and their trajectories from takeoff (at *t*≈−1.5 s) are designated by yellow and red lines. (B) An image taken in the dark at *t*=500 ms, after the bees had hit the bottom of the container, with the bees' positions marked by circles. The black dot on each trajectory marks the bee's position at *t*=0. Dark yellow and dark red lines mark the bees' trajectories at *t*<0. Yellow and red dotted lines mark the bee's trajectories at *t*>0, where dots show per-frame positions. The response times of the two bees were 116 ms and 150 ms. (C) The probability distribution of the bees' response time to the onset of darkness, calculated over 217 events. The response time is defined as the time where an observable trajectory change was detected. The mean±s.d. of the response was 98.4±39.4 ms, and is designated by thick and thin dashed vertical lines.

To analyze the bee data, we manually measured their response time to the onset of darkness. The response time was defined as the time from *t*=0 until a change in the trajectory was observed. Because the changes of the bees' trajectories upon darkness were large enough to be manually detected, we estimated the accuracy of the response time to be ±16 ms (±1 video frame). All dark perturbation events ended in a crash either to the bottom or to the sidewall of the chamber. In the constant darkness experiment, we verified that no bee took off in the dark. Sample trajectory data (Fig. 2) were obtained by manually tracking the positions of both bees.

### Definition of velocities and the angle γ

We define *V*_fwd_ as the velocity component in the *xy* plane projected onto the insect's forward-facing axis 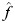 (Fig. 1B):
(1)

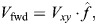
where *V_xy_* is the insect's horizontal velocity, namely, its center-of-mass velocity vector projected onto the *xy* plane in the lab frame. To quantify the angular change in the velocity direction, we defined the angle γ as the angle between the horizontal velocity at time *t* and that at time *t*=0:
(2)

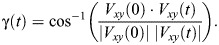
The cone angle α_cone_, plotted in Fig. S2A, is defined as the angle between the insect's center-of-mass velocity vector *V* in 3D and 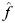:
(3)

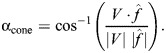


### Analysis of acceleration direction

**Fig. 3.**
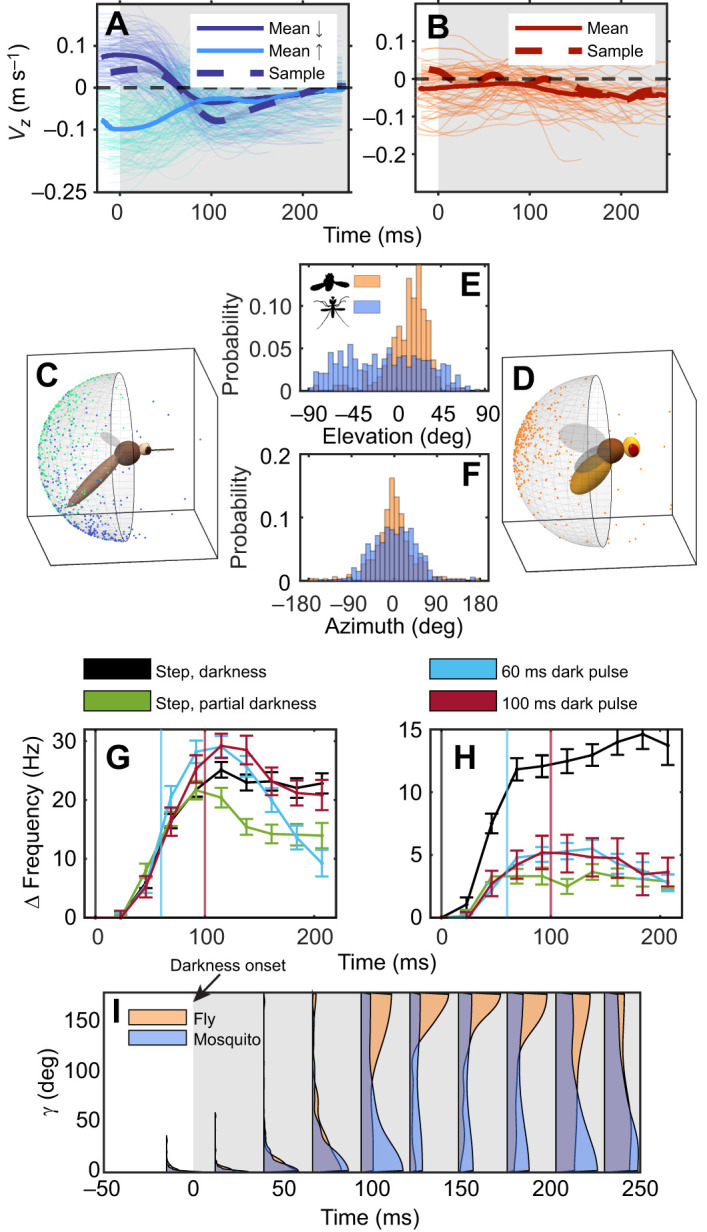
**Kinematics of the mosquito and fruit fly responses to a darkness step perturbation.** (A,B) Vertical velocities for all step perturbations for mosquitoes (A) and fruit flies (B), with means (solid bold lines) and representative events (dashed lines). In A, up and down arrows indicate the direction of the mosquitoes' initial vertical velocity. (C–F) The direction of maximum acceleration in the insects' body frame during the dark response maneuver: a spherical scatter for mosquitoes (C) and flies (D); azimuth (E) and elevation angle (F) distributions of the maximum acceleration direction for both species. (G,H) Mean wingbeat frequency difference with respect to pre-perturbation values, under various perturbations for the mosquito (G) and fly (H). (I) Distribution of γ – the change of horizontal flight direction – in all step perturbation events (grayed area) for both species at distinct times.

The insect's center-of-mass acceleration was calculated as detailed above and then represented in the body frame-of-reference as *a*=[*a_x_*(*t*), *a_y_*(*t*), *a_z_*(*t*)]. To find the direction of maximum acceleration in the body frame (Fig. 3), we first calculated the direction of the body accelerations *t*_acc_=70 ms for the mosquitoes and *t*_acc_=90 ms for the fruit flies. The acceleration direction was represented by its azimuth φ_acc_ and elevation θ_acc_ angles:
(4)

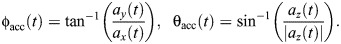


### Acceleration-based response threshold

To estimate the response time (RT) of each species to the onset of darkness, we defined a threshold as follows. First, for each species, we calculate the nominal acceleration distribution, which consists of the pre-perturbation acceleration norm across all events. Second, we set the acceleration-based response threshold for response to be 3 standard deviations above the mean of this distribution. Finally, for each event, the response time was identified as the time when the acceleration norm passed this threshold. The remaining features by which the responses were analyzed are defined in the Results.

### Statistical analysis of dark responses with cut aristae

To perform Fisher’s exact tests for comparing intact insects and insects with both arista removed, we used the function fishertest in MATLAB.

### Unit conversion to light illuminance in lux

To convert from W cm^−2^ to lx (illuminance) we used the conversion factor for a 555 nm wavelength: 1 W cm^−2^=683 lx. To compare light levels reported in previous work in units of cd m^−2^ (luminance), we converted to lux by assuming that the light source is a full hemisphere of a perfectly diffusive emitter (a Lambertian source), representing the overhead sky. The relationship between illuminance and luminance is given by:
(5)

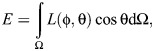
where *E* is in lux and the luminance *L* (cd m^−2^) is integrated over the solid angle Ω. Under the assumption of constant luminance, we obtained *E*=π*L*.

## RESULTS

To measure the response of free-flying fruit flies and mosquitoes to sudden darkness, we recorded their flight using four high-speed cameras in a light-controlled environment (Maya et al., 2023; Ben-Dov and Beatus, 2022) and under infrared illumination undetectable by these species (Sharkey et al., 2020; Gibson, 1995; Yamaguchi et al., 2010; Muir et al., 1992) (Fig. 1A–C; see Materials and Methods; Movies 1–3). In these experiments, the ambient light was typically on at 34 lx illuminance, and in each flight event, the light was automatically and abruptly turned off at *t*=0, resulting in a 500,000-fold illuminance reduction to 6.8×10^−5^ lx, which we term ‘darkness’ (see Materials and Methods). We conducted two types of these perturbation experiments: in a step perturbation (Movie 1), darkness persisted after *t*>0 during the entire flight event and the light was turned back on long after the end of the recording, while in a pulse perturbation (Movie 2), the light was turned back on after a predetermined time of 5, 20, 40, 60, 80 or 100 ms. Flight kinematics were extracted for each event (see Materials and Methods). Because such a sudden and large illuminance reduction is a perturbation that these animals probably do not meet in the wild, we also measured the mid-air responses of fruit flies and mosquitoes to partial darkness perturbations (Movie 3), where illuminance decreased 10-fold, from 34 to 3.4 lx. This perturbation mimics more realistic scenarios; for example, a transition from a sunlit to a shaded area. As control experiments, we measured flight under either constant light or constant darkness. In addition, to compare the flight in the dark of these Diptera with the flight of another insect order, we measured the flight of honey bees (*Apis mellifera*, order: Hymenoptera), in three conditions: in continuous light (2600 lx), in continuous darkness (9.6×10^−4^ lx) and under sudden darkness perturbations, in which the light was abruptly reduced from the continuous level to the darkness (9.6×10^−4^ lx) level (see Materials and Methods; Movie 4).

### Fruit flies and mosquitoes fly in constant darkness while honey bees do not

We measured the flight of fruit flies and mosquitoes under continuous darkness (6.8×10^−5^ lx; see Materials and Methods), where they had sufficient time to become dark adapted. Both species were able to take off and fly stably in continuous darkness, though they did so much less frequently than in the light: in the dark, fruit flies and mosquitoes took off 15 times and 6 times less often than in lit conditions, respectively (Table 1; Movies 5 and 6). These ratios are based on the number of flight events per unit time recorded in the light (34 lx) and under continuous darkness, in the same experiment with the same number of animals. Flight trajectories in continuous darkness were qualitatively similar to trajectories in the light, where the main difference was that fruit flies were flying with their legs extended rather than folded in. Importantly, these insects exhibited stable flight, e.g. straight or ascending, with no signs of instability or impeded controllability. To fly into the filming volume, they had to take off and maintain controlled flight until they reached the middle of the experimental chamber, 10 cm away from its boundaries. Moreover, the duration of the recorded trajectories, ∼250 ms (55 fruit fly wingbeats and 155 mosquito wingbeats) was significantly longer than the characteristic instability time of flies (17 wingbeats; Perl et al., 2023) and mosquitoes (50 wingbeats; Liu and Sun, 2019), indicating that their flight in the dark condition had to employ active control to maintain stability. The flight behavior of the honey bees was different: in the light (2600 lx) they were flying continuously but in the dark (9.6×10^−4^ lx) we did not observe a single flight event (1 h observation time on ∼30 individuals in each condition; see Materials and Methods).

As a reference for the Diptera darkness response, we compared the overall flight characteristics and body morphology of *D. melanogaster* and *C. pipiens* (Fig. 1B,C, Table 1). Both dipterans weigh ∼1 mg, and mosquitoes have longer, more slender bodies and longer extended legs compared with fruit flies. Honey bees, for comparison, weigh 116±30 mg (Vance et al., 2014). Further, mosquito wings have a higher aspect ratio than fruit fly wings (Fig. 1C) and we measured that the mosquito wingbeat frequency is ∼3 times higher (*f*_mos_=655±42 Hz, *f*_fly_=233±13 Hz) with a much smaller peak-to-peak amplitude (Bomphrey et al., 2017) (*A*_mos_=44 deg, *A*_fly_=142 deg). For *A. mellifera*, the frequency and amplitude are 227±13 Hz and 87±8 deg, respectively (Vance et al., 2014). The velocity distributions of mosquitoes and fruit flies show that *C. pipiens* fly faster than *D. melanogaster*, and while fruit flies tend to fly mostly forward and horizontally, the mosquitoes exhibit a significant vertical and sideways motion (Fig. S1A,B).

### Upon sudden darkness, fruit flies and mosquitoes exhibit a similar response and continue flying, while bees crash down

The kinematic response of mosquitoes and fruit flies to a darkness step perturbation is shown in Fig. 1D–I and Movie 1. This response was not observed in any of the control experiments (Fig. S1), which included both unperturbed flight events (Table 1) and the pre-perturbation parts of the experiment (from *t*=−40 ms to *t*=0 in each flight event). Representative flight trajectories of both species (Fig. 1D,E) show marked changes of both direction and velocity starting at *t*=30–40 ms. In both events, the insects increased their body pitch angle (Fig. 1B,F,H) and slowed down their forward horizontal velocity, *V*_fwd_ (Fig. 1G,I). This combined motion of pitching up and slowing down is consistent with the helicopter model for velocity control (Vogel, 1966). According to this model, similar to the way in which a helicopter moves backwards (forward) by pitching up (down) to redirect its lift vector, insects modulate their body pitch angle to redirect their mean lift vector to control forward velocity. By *t*≈80 ms, both insects reached a full stop of *V*_fwd_=0 and started flying backwards. Subsequently, both insects flew forward again in a different direction from their direction at *t*=0. To characterize the typical response maneuvers of these species, we analyzed 280 mosquito responses (Fig. 1F,G) and 62 fruit fly responses (Fig. 1H,I), and calculated their mean kinematics and timing statistics (Fig. 1J–L). As we show below, examining the mean kinematics showed that in both species, the response followed a consistent sequence: pitching up and slowing down of *V*_fwd_, increasing wingbeat frequency and changing course.

During this response sequence, both fruit flies and mosquitoes continued to fly in a persistent and controllable manner at least within the 250 ms recorded time window after the onset of darkness. To compare this behavior with the response of an insect of a different order, we measured the response of honey bees under a similar perturbation (see Materials and Methods). In contrast to the two dipterans, honey bees persistently crashed down immediately after the onset of darkness in all of the 217 recorded events (Fig. 2A,B; Movie 4) (Faust, 1952). We observed that 98±39 ms after the onset of darkness, the bees changed their trajectory by pitching down and moving downwards, which resulted in them either hitting the bottom of the container or colliding with its sidewall (Fig. 2C). In some cases, we observed the bees tumbling about their pitch axis, which hints that their motion was uncontrolled. As the honey bees consistently crashed in the dark and fruit flies and mosquitoes did not, we next focused on the common and different features of the darkness responses in these two dipterans.

#### Pitching up and braking

In both fruit flies and mosquitoes, the response started with a pitch-up maneuver (Fig. 1F,H) of ∼10 deg on average that induced braking of *V*_fwd_, consistent with the helicopter model. Fig. 1G,I illustrates the braking of *V*_fwd_ associated with the increase in body pitch. The response times (RT; Fig. 1J), defined using an acceleration threshold (see Materials and Methods), were 43±18 ms for the mosquitoes and 48±13 ms for the fruit flies (Fig. 1J), where 79% of the mosquitoes and 84% of the flies qualified for the response criterion. These response times are similar to the visual-motor response time known for flies (Muijres et al., 2014; Land and Collett, 1974; Bender and Dickinson, 2006; Card and Dickinson, 2008). Subsequently, although both species pitched up to slow down their flight velocity, their mean kinematics were different. For the fruit flies, in 74% of events they achieved a full stop of *V*_fwd_ with a mean braking time of 85±14 ms (Fig. 1K) and then, on average, started flying backwards (Fig. 1I). Yet, in the mosquitoes, only 49% of the trajectories stopped fully in *V*_fwd_, with a mean braking time of 96±35 ms, and with no backwards flight in the mean trajectory. In the vertical direction, this trend reversed: more mosquito trajectories (70%) got to a full stop of their vertical velocity *V_z_*, compared with 56% of the flies' trajectories. In both species, the mean vertical stop time was *t*≈70 ms (Fig. 1L). Dividing the mosquito trajectories according to their initial vertical flight direction showed that trajectories flying initially upwards (*V_z_*>0) responded by accelerating downwards, and vice versa (Fig. 3A). The flies' trajectories did not show this trend (Fig. 3B).

Overall, the form of velocity braking of mosquitoes and fruit flies is consistent with their nominal flight behavior, in which mosquitoes exhibit more vertical motion and flies tend to fly more horizontally (Fig. S2). Fig. 3C–F shows the direction of body accelerations taken at *t*=70 ms for the mosquitoes and *t*=90 ms for the fruit flies, which are the mean times of maximum acceleration magnitude for each species. For the flies, the maximum acceleration is consistently oriented backwards with respect to the fly, whereas for the mosquitoes, the direction of acceleration is more widely distributed: in the azimuthal direction (yaw in the lab frame of reference) body acceleration is centered towards the back and more uniformly distributed in the elevation direction (pitch in the lab frame of reference).

#### Increasing wingbeat frequency

In parallel with pitching up and braking of *V*_fwd_, both species significantly increased their wingbeat frequency by ∼5% with respect to the pre-perturbation value (Fig. 3G,H; *P*<3×10^−4^ at *t*≥60 ms). This increase can compensate for the loss of vertical force due to pitching up, consistent with the helicopter model. Notably, once the light turned back on after the pulse perturbations, the wingbeat frequency of both species started to decrease back to its pre-perturbation value.

#### Change of flight direction

To quantify the change of flight direction during the maneuver, we define the angle γ, which is the angle between the horizontal velocity directions at time *t* and at time 0 (see Materials and Methods). Fig. 3I shows the distributions of γ at distinct times for all step perturbations. For example, if γ is concentrated around 0, it implies that the insects flight direction is approximately aligned with their initial direction at *t*=0, and when γ is concentrated around 180 deg, they fly in the opposite direction. On average, both species maintained their initial flight direction until *t*≈50–70 ms, after which their directional response differed. Between *t*=100 and *t*=175 ms flies generally flew backwards, while the mosquitoes exhibited a uniformly distributed flight direction. By the end of the maneuver, at *t*≥175 ms, both species flew in an almost uniformly distributed direction, with a slight tendency of the mosquitoes to regain their pre-perturbation direction. Examining the body roll dynamics in the flight trajectories both species, we did not observe a distinct trend across the dataset (Fig. S5A,B). Yet, the roll angle was correlated with the change in yaw angle with respect to *t*=0, indicating that both species were performing coordinated turns (Maya et al., 2023) (Fig. S5C,D).

#### Leg extension

Finally, a notable feature of the flies' response, which was absent in mosquitoes, is extension of their legs (Movies 1, 2 and 7). Mosquitoes flew with their legs constantly extended, regardless of the light conditions. In 74 of the 76 fly responses to step perturbations (97.3%), they extended their legs at *t*=52±15 ms on average, which overlaps with their deceleration-based response time. In one of these events, the fly entered the filming volume with its legs already extended. Leg extension increases the fly's moment of inertia and aerodynamic drag and, thus, increases its robustness to external perturbations, similar to bees flying in turbulent air (Combes and Dudley, 2009). We hypothesized that leg extension may be associated with a pre-landing or pre-impact response (Van Breugel and Dickinson, 2012). Interestingly, in our continuous darkness experiments, where fruit flies were both taking off and flying in the dark, they were also flying with their legs extended (Movie 5), but during the previously reported mid-air looming response, their legs were kept folded (Muijres et al., 2014).

Collectively, these results suggest that the flies' and mosquitoes' response to sudden darkness is a well-defined and reproducible maneuver, with very similar characteristics among the two species. The responses to short dark-pulse perturbations in fruit flies and mosquitoes have a similar onset but different orientation recovery.

#### Orientation recovery

**Fig. 4.**
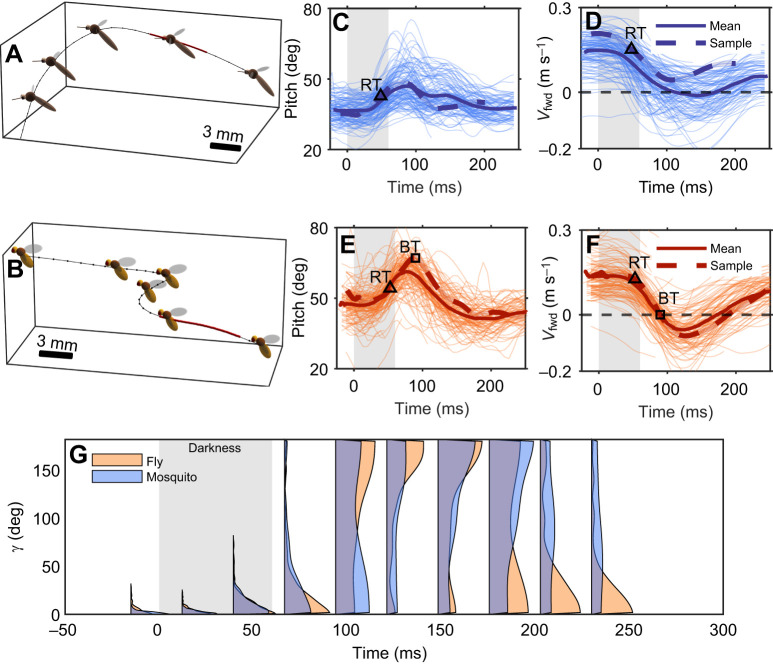
**Response to a 60** **ms dark pulse perturbation shows a similar onset but different orientational recovery in flies and mosquitoes.** (A,B) Representative 3D trajectories of a mosquito (A) and a fly (B); the trajectory is marked in red during darkness. (C–F) Pitch angle (left) and forward velocity (right) for mosquitoes (C,D) and flies (E,F) (means, solid bold lines; and representative events, dashed lines). RT, response time; BT, brake time (*V*_fwd_=0) are marked for the representative events. (G) Distribution of γ across all 60 ms ‘pulse’ perturbations (grayed area) for both species at distinct times.

To further characterize the insects' response to sudden darkness and their response threshold, we measured the flight trajectories of both species under short dark pulses of 60 and 100 ms (see Materials and Methods). Fig. 4A,B and Movie 2 show representative trajectories of a mosquito and a fruit fly responding to a 60 ms pulse. The first stages of these maneuvers – the coupled pitching up and slowing down, as well as the increase in wingbeat frequency – are similar to the step response. Yet, the subsequent directional response in these events is different. The fruit fly first changed direction to move backwards, as in the step response, but later returned towards its initial flight direction, recovering its overall initial direction by *t*=250 ms. The mosquito, in contrast, did not recover its initial direction.

**Fig. 5.**
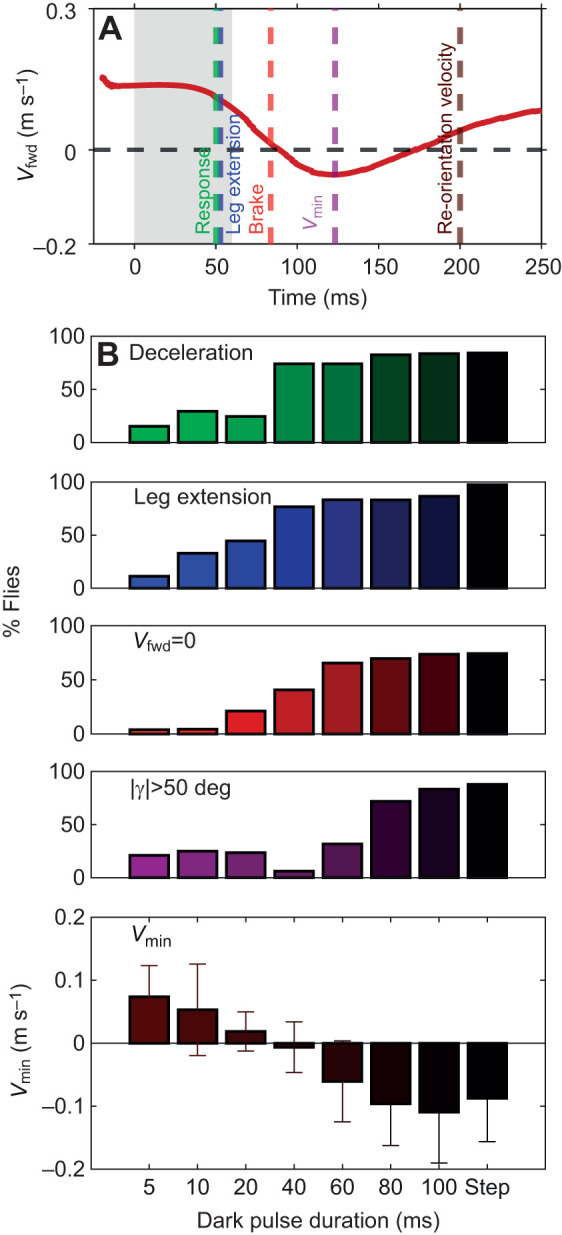
**Timeline and response rates of the flies’ dark response for different dark pulse durations.** (A) General timeline of the characteristic fruit fly response, plotted for the 60 ms perturbation. (B) Top: the percentage of fruit flies responding to various dark-pulse durations according to four criteria: deceleration, leg extension, braking *V*_fwd_ and direction loss γ<50 deg at *t*=200 ms. Bottom: the minimum value of *V*_fwd_ between 15 and 150 ms. Whiskers represent one standard deviation for each type of perturbation. In all panels, a step perturbation implies that darkness persists past the end of the recorded event.

Examination of the entire dataset of 132 mosquito and 119 fly maneuvers shows that the beginning of both responses was similar to the step response, but after the light was turned back on, the responses differed (Fig. 4C–F). While in step perturbations, mosquitoes remained at a high body pitch angle, in 60 ms pulse perturbations, after the light was turned back on, they started to pitch down towards their pre-perturbation pitch angle. Similarly, flies on average returned to their pre-perturbation pitch angle faster than in step perturbations. Additionally, both species, on average, increased *V*_fwd_ to positive values at *t*>200 ms. In the end of the maneuver, the most apparent difference between the species' responses was in the distribution of their flight direction angle γ (Fig. 4G). While the mosquitoes' directional distributions were overall similar to their response under step perturbations – changing direction to eventually fly in an almost random direction – the flies at *t*>160 ms consistently returned to their overall pre-perturbation direction within a standard deviation of 36 deg at 230 ms, corresponding to the end of the recorded events. These features were also observed in 100 ms dark pulses experiments (Fig. S3). The timeline of the flies’ dark response under a 60 ms pulse is shown in Fig. 5A. The mosquitoes' response was similar, without leg extension and velocity reorientation.

#### Fruit fly response rate decreases as pulse duration decreases

Next, we focused on the effect of dark pulse duration on the fruit flies, testing their response to 5, 10, 20, 40, 60, 80 and 100 ms pulses. For each pulse duration, we calculated the percentage of events in which fruit flies responded to the perturbation according to several criteria (Fig. 5B): the deceleration criterion used above, leg extension, fully braking to *V*_fwd_=0, and loss of initial flight direction γ>50 deg at *t*=200 ms. Additionally, we quantified velocity braking by calculating the minimum *V*_fwd_ between 15 and 150 ms. Consistent across all criteria, the percentage of fruit fly responses and their minimum *V*_fwd_ decreased as the dark pulse duration decreased, with a marked decrease for dark pulses shorter than 40 ms. Yet, some flies did respond even to the shortest 5 ms pulses. Further, this temporal sensitivity experiment shows that the fruit flies' behavioral sequence (Fig. 5A) was interrupted after the light was turned back on.

### The aristae are not essential for flight in the dark

It is difficult to know what visual information the flies and mosquitoes extract under the dark condition in our experiment, yet it has been shown that the light adaptation time in fruit flies is of the order of 1 s, longer than our post-darkness observation time of ∼250 ms (Ketkar et al., 2023). Assuming that during this timeframe the fruit flies and mosquitoes have not yet adapted to the dark condition and have no access to visual cues, here we asked whether their flight control system uses sensory information from the antennae to compensate for this temporary loss of vision; for example, by measuring the direction of gravity.

It has been previously shown that during flight, fruit flies use their antennae to measure their airspeed (Fuller et al., 2014; Mamiya and Dickinson, 2015; Suver et al., 2019). When walking, they can sense the direction of gravity using their halteres (Daltorio and Fox, 2018) and antennae (Horn and Lang, 1978; Kamikouchi et al., 2009; Kladt and Reiser, 2023 preprint), and by measuring contact forces with their legs (Horn and Lang, 1978; Agrawal et al., 2020). As contact forces are irrelevant in free flight and as the haltere sensitivity is believed to be too low to measure the acceleration of gravity in free flight (Nalbach, 1993; Hengstenberg, 1988), we asked whether flies and mosquitoes use their antennae for this task. As it has been shown that flies whose aristae – a bristled structure at the distal part of the antenna – have been ablated do not exhibit normal gravitaxis (Kamikouchi et al., 2009), we measured the darkness response of both species after removing their two aristae (see Materials and Methods). Previous studies have shown that removing the aristae diminishes the flies' auditory responses (Manning, 1967; Von Schilcher, 1976), and has a minor effect on the flapping patterns in tethered experiments (Duistermars et al., 2009). Yet, the free flight of fruit flies or mosquitoes with damaged antennae has not been documented. We compared the darkness responses of aristae-ablated fruit flies and mosquitoes with those of intact insects by counting the number of flight events that exhibited a dark response according to the criteria used above in Fig. 5B. Overall, both species were able to fly in the dark condition after arista ablation. As shown in Tables 2 and 3, without the aristae, the flies' response rates significantly decreased in all criteria (*P*<5.4×10^−3^), whereas the mosquitoes’ response rates did not change significantly (*P*=0.18 to 0.78).

**Table 2.** The number of recorded fruit fly responses to sudden darkness, with intact antenna and with cut aristae, according to four response criteria

Fruit flies	Intact	No aristae	*P*-value
Deceleration response	89 (84%)	33 (61%)	<0.001
No deceleration response	17 (16%)	21 (39%)	
Extended legs	184 (91%)	80 (77%)	<0.001
Did not extend legs	19 (9%)	24 (23%)	
Full stop of *V*_fwd_=0	88 (63%)	46 (44%)	<0.001
No full stop of *V*_fwd_=0	52 (37%)	58 (56%)	
Full stop of *V_z_*=0	67 (56%)	18 (33%)	5.4×10^−3^
No full stop of *V_z_*=0	52 (44%)	36 (67%)	

*P*-values were calculated using Fisher's exact test. Here, ‘step’ and 100 ms ‘pulse’ perturbations were pooled together. The leg extension statistics also include events that have been analyzed manually.

**Table 3.** The number of recorded mosquito responses responding to sudden darkness, with intact antenna and with cut aristae, according to three response criteria

Mosquitoes	Intact	No aristae	*P*-value
Deceleration response	220 (79%)	84 (77%)	0.78
No deceleration response	60 (21%)	25 (23%)	
Full stop of *V*_fwd_=0	137 (49%)	48 (44%)	0.43
No full stop of *V*_fwd_=0	143 (51%)	61 (56%)	
Full stop of *V_z_*=0	195 (70%)	68 (62%)	0.19
No full stop of *V_z_*=0	85 (30%)	41 (38%)	

*P*-values were calculated using Fisher's exact test.

### In fruit flies and mosquitoes, the partial darkness response is similar to the darkness response

It is plausible that an abrupt 500,000-fold reduction of illuminance from 34 to 6.8×10^−5^ lx is not a naturally occurring stimulus that fruit flies and mosquitoes have adapted to. To test whether the darkness response maneuver is also performed in a biologically relevant scenario, we measured the mid-air response of these insects under a partial-darkness perturbation, where light intensity decreased 10-fold with respect to the same lit conditions as before (Movie 3). This perturbation mimics, for example, an in-flight transition from a sunlit area to a shaded area under tree canopy. We recorded 378 mosquito flight events and 86 events for fruit flies. Applying the same analyses as above showed that partial darkness induced a maneuver that is mostly similar to the darkness response shown above (Fig. 3G,H; Fig. S4). The responses in both species included a similar increase in wingbeat frequency (Fig. 3G,H) and a similar pitching-up motion (Fig. S4B,C), which induces braking of their forward speed (Fig. S4D,E). The acceleration-based response rates and response times were almost identical under the two perturbations (Fig. S4F): according to the acceleration criterion, 83% of the flies and 77% of the mosquitoes responded to partial darkness, compared with 84% and 78% response rates under complete darkness, respectively (Fisher’s exact test *P*=0.83 for the fruit flies and *P*=0.79 for the mosquitoes). The mean acceleration-based response times under partial darkness were 46 ms for the flies and 39 ms for the mosquitoes, compared with 48 ms and 43 ms, respectively, under the darkness perturbation. Similar trends also appeared in the *V*_fwd_ brake time and *V_z_* brake time (Fig. S4G,H). The directional distribution of maximum acceleration as well as the distribution of γ, the horizontal change of the flight direction, were also similar between the responses to both perturbations (Fig. S4I–M).

In parallel with these similarities, we characterized three differences between the responses to darkness versus partial darkness. First, while in the end of the darkness response, after ∼250 ms, both fruit flies and mosquitoes flew in an almost random direction with respect to their initial direction (Fig. 3I), under partial darkness, the distribution of flight direction γ for both species showed a preference for flight in the initial direction (Fig. S4M). In fruit flies, the directional distribution at the end of the partial darkness maneuver was similar to their response to darkness pulse perturbations. Second, under partial darkness, the fruit fly leg extension rate was 73.3% compared with 97.3% under the darkness perturbations (Fisher’s exact test *P*<0.001). And, third, a minor difference between the responses to the two perturbations was seen in mosquitoes: in the darkness perturbations, 49% of the events exhibited a full stop of *V*_fwd_, while in partial darkness, a full stop appeared in only 20% of the events (Fisher’s exact test *P*<<0.001). Overall, these data show that in both species, the responses to darkness and partial darkness step perturbations are similar in most features, particularly during the response onset, but they show different final distributions of the flight direction.

## DISCUSSION

In this work we compared the flight of fruit flies, mosquitoes and honey bees by testing how they handle flight in either constant or sudden darkness. The differences in the observed behaviors, especially between the honey bees and the two dipterans, are likely attributed to differences in their sensory modalities: their visual systems, and whether or not they have halteres. First, flies and mosquitoes have visual specializations (superposition eyes in fruit flies, broad rhabdoms in mosquitoes) that endow them with highly sensitive eyes, in contrast to the significantly less sensitive apposition eyes of honey bees. Second, like all Diptera, fruit flies and mosquitoes have halteres, while honey bees do not. Importantly, the flight of many insect species, including these three species, is inherently unstable in both the longitudinal and lateral axes (Sun, 2014; Perl et al., 2023; Liu and Sun, 2019; Xu and Sun, 2013), which requires constant and rapid control actions by the wings. For example, the response time of fruit flies to mid-air roll perturbations is a single wingbeat (∼5 ms) (Beatus et al., 2015). Such short latency is essential for controlling their lateral instability (Perl et al., 2023). In Diptera, the primary sensor for these rapid stabilization mechanisms is believed to be the halteres. In the fruit fly, our current controller models for yaw (Ristroph et al., 2010), pitch (Whitehead et al., 2015, 2022) and roll (Beatus et al., 2015) rely on integration of the haltere angular velocity signal to obtain the body angular attitude. Such temporal integration, also known as ‘dead reckoning’, is prone to drift as a result of the accumulation of measurement errors. Yet, the magnitude and dynamics of this hypothesized haltere-integration drift are unknown. Based on the duration of fruit fly correction maneuvers, we can only estimate that haltere integration is successfully used for flight control at least within tens of milliseconds, a timeframe in which the halteres are most probably the dominant sensor in the flight control pathway. The low-light sensitivity of the bees and the fact they do not have halteres to sense their body angular velocity can explain why, consistent with previous work, honey bees do not fly in the dark, and explains our observation that upon sudden mid-flight darkness they seem to stop flying and crash within ∼100 ms. It is likely that as the ambient light level drops below their sensitivity threshold and as we (still) do not know of any other sensory modality they use to estimate their angular attitude, they cease to fly upon darkness, either voluntarily or involuntarily owing to their inherent instability.

In contrast, fruit flies and mosquitoes were able to voluntarily take off and maintain stable flight under continuous darkness (6.8×10^−5^ lx), though our experimental setup could test their performance in a limited way: they were able to reach the filming volume, 10 cm away from the chamber boundary, and maintain a seemingly stable flight for ∼250 ms during their passage through this volume. In addition, their takeoff events were less frequent than under lit conditions. It is likely that under continuous darkness, the flies and mosquitoes had been fully light adapted, yet it is unknown what visual information they are able to extract under these conditions. Their capability to voluntarily fly under these conditions may indicate that, when light adapted, they are able to extract sufficient visual information during flight. Alternatively, it may be that they fly without visual cues, relying mostly on their halteres for flight stabilization. Interestingly, the observation by Leonte et al. (2021) that in the dark fruit flies fly faster than under lit conditions, may appear opposite to the tradeoff of insect flight at low light, where longer light-integration time leads to slower flight speeds to reduce motion blur. Possible explanations may arise from future experiments that would explore what visual information these animals can behaviorally exploit under low light. Behaviorally, this would entail measuring flight kinematics during longer post-darkness times and at light levels significantly lower than the insects' sensitivity thresholds.

Upon a sudden reduction of light intensity in mid-air from lit conditions (34 lx) either by 500,000-fold (to 6.8×10^−5^ lx) or by 10-fold to partial darkness (3.4 lx), both fruit flies and mosquitoes continued flying at least within the ∼250 ms post-perturbation observation time. During this timeframe, the two dipterans exhibited a similar and well-defined response sequence. The sequence consists of a visual-motor delay of ∼45 ms, increasing the wingbeat frequency, pitching up, slowing down and changing course (backwards and then random orientation under complete darkness, backwards/braking and then grossly regaining orientation under partial darkness). The non-directional response to darkness is qualitatively different from the looming response in fruit flies (Muijres et al., 2014) and mosquitoes (Cribellier et al., 2022, 2024), which is a directional response implemented by a banked avoidance turn away from the stimulus and, in fruit flies, does not include leg extension. It is reasonable that the slowing down of fruit flies and mosquitoes in response to sudden darkness and sudden partial darkness is consistent with the tradeoff between the increase in photoreceptor integration time to collect more light and the reduction of flight speed to reduce motion blur. Additionally, flies consistently extended their legs, which increases their moment of inertia and thus increases their resilience to mechanical perturbations (Combes and Dudley, 2009), and may also be part of a landing (Van Breugel and Dickinson, 2012) or pre-impact response. Changing of the wingbeat frequency in response to darkness raises questions regarding the resonant nature of the dipteran flight motor system, and to what extent it can maintain energetic efficiency while applying frequency modulation (Pons and Beatus, 2022, 2023; Pons et al., 2023; Pons, 2023).

As the dynamics of the light adaptation process in fruit flies and mosquitoes is only partially understood, it is difficult to assess when and to what extent these insects light adapt following the sudden darkness perturbation applied here. Assuming an adaptation time of the order of 1 s (Ketkar et al., 2023) implies that during our observation timeframe of ∼250 ms, the insects may not be fully adapted to the dark. Further electrophysiological measurements are required to understand light adaptation under these conditions. It is likely that the rapid, haltere-mediated stabilization reflexes of fruit flies and mosquitoes increase their probability of remaining aloft long enough to mitigate such sudden darkness events in a natural behavioral context. This ‘extra’ airborne time could be used for light adaptation, performing a safe landing, or for flying to a brighter region of space, as demonstrated by the recovery following dark-pulse perturbations.

Aristae ablation reduced the darkness response rate in fruit flies but not in mosquitoes, which may indicate a different role of the antennae in the flight control mechanisms of these two species. The responses to short, 60 ms and 100 ms, dark-pulse perturbations in fruit flies and mosquitoes had a similar onset but different orientation recovery, where fruit flies tended to regain their velocity direction while mosquitoes ended up flying in a random direction, as in their response to step perturbations. These different behaviors may point to a different capacity of these two species to adapt to the transition from darkness back to the lit condition. In fruit flies, there have been events where animals initiated a dark-response maneuver even after 5 ms dark pulses, which can set a limit for their visual integration time.

Free-flight experiments under controlled light conditions with mid-air mechanical perturbations (Ristroph et al., 2010; Vance et al., 2013; Jakobi et al., 2018), combined with further electrophysiological characterization of insect light sensitivity and adaptation, would be a promising route towards understanding the dynamic role of vision in flight, the neural encoding of direction (Green et al., 2017; Lyu et al., 2022), gravity sensing (Goulard et al., 2016, 2018; Daltorio and Fox, 2018; Horn and Lang, 1978; Kamikouchi et al., 2009; Kladt and Reiser, 2023 preprint; Hengstenberg, 1993; Kendzel et al., 2023; Walthall and Hartman, 1981) and mid-air sensory fusion (Taylor and Krapp, 2007; Verbe et al., 2023; Fuller et al., 2014; Suver et al., 2019; Fabian et al., 2024).

## Supplementary Material

10.1242/jexbio.251675_sup1 Supplementary information

## References

[JEB251675C1] Agrawal, S., Dickinson, E. S., Sustar, A., Gurung, P., Shepherd, D., Truman, J. W. and Tuthill, J. C. (2020). Central processing of leg proprioception in *Drosophila*. *eLife* 9, e60299. 10.7554/eLife.60299 33263281 PMC7752136

[JEB251675C2] Anwar, M. Z., Tobalske, B. W., Agrawal, S., Mongeau, J.-M., Luo, H. and Cheng, B. (2024). Hummingbirds rapidly respond to the removal of visible light and control a sequence of rate-commanded escape manoeuvres in milliseconds. *Proc. B* 291, 20241268. 10.1098/rspb.2024.1268 PMC11576114 39561797

[JEB251675C3] Baird, E., Fernandez, D. C., Wcislo, W. T. and Warrant, E. J. (2015). Flight control and landing precision in the nocturnal bee *Megalopta* is robust to large changes in light intensity. *Front. Physiol* 6, 305. 10.3389/fphys.2015.00305 26578977 PMC4623526

[JEB251675C4] Bar, N. S., Skogestad, S., Marçal, J. M., Ulanovsky, N. and Yovel, Y. (2015). A sensory-motor control model of animal flight explains why bats fly differently in light versus dark. *PLoS Biol.* 13, e1002046. 10.1371/journal.pbio.1002046 25629809 PMC4309566

[JEB251675C5] Beatus, T., Guckenheimer, J. and Cohen, I. (2015). Controlling roll perturbations in fruit flies. *J. R. Soc. Interface* 12, 20150075. 10.1098/rsif.2015.0075 25762650 PMC4387536

[JEB251675C6] Bender, J. A. and Dickinson, M. H. (2006). Visual stimulation of saccades in magnetically tethered *Drosophila*. *J. Exp. Biol.* 209, 3170-3182. 10.1242/jeb.02369 16888065

[JEB251675C7] Ben-Dov, O. and Beatus, T. (2022). Model-based tracking of fruit flies in free flight. *Insects* 13, 1018. 10.3390/insects13111018 36354842 PMC9692569

[JEB251675C8] Bomphrey, R. J., Nakata, T., Phillips, N. and Walker, S. M. (2017). Smart wing rotation and trailing-edge vortices enable high frequency mosquito flight. *Nature* 544, 92-95. 10.1038/nature21727 28355184 PMC5412966

[JEB251675C9] Borst, A. and Groschner, L. N. (2023). How flies see motion. *Annu. Rev. Neurosci.* 46, 17-37. 10.1146/annurev-neuro-080422-111929 37428604

[JEB251675C10] Card, G. and Dickinson, M. H. (2008). Visually mediated motor planning in the escape response of *Drosophila*. *Curr. Biol.* 18, 1300-1307. 10.1016/j.cub.2008.07.094 18760606

[JEB251675C11] Combes, S. A. and Dudley, R. (2009). Turbulence-driven instabilities limit insect flight performance. *Proc. Natl Acad. Sci. USA* 106, 9105-9108. 10.1073/pnas.0902186106 19458254 PMC2690035

[JEB251675C12] Cribellier, A., Straw, A. D., Spitzen, J., Pieters, R. P. M., van Leeuwen, J. L. and Muijres, F. T. (2022). Diurnal and nocturnal mosquitoes escape looming threats using distinct flight strategies. *Curr. Biol.* 32, 1232-1246.e5. 10.1016/j.cub.2022.01.036 35134328

[JEB251675C13] Cribellier, A., Camilo, L. H., Goyal, P. and Muijres, F. T. (2024). Mosquitoes escape looming threats by actively flying with the bow wave induced by the attacker. *Curr. Biol.* 34, 1194-1205. 10.1016/j.cub.2024.01.066 38367617

[JEB251675C14] Currea, J. P., Frazer, R., Wasserman, S. M. and Theobald, J. (2022). Acuity and summation strategies differ in vinegar and desert fruit flies. *iScience* 25, 103637. 10.1016/j.isci.2021.103637 35028530 PMC8741510

[JEB251675C15] Daltorio, K. A. and Fox, J. L. (2018). Haltere removal alters responses to gravity in standing flies. *J. Exp. Biol.* 221, jeb181719. 10.1242/jeb.181719 29853546

[JEB251675C16] Dickinson, M. H. and Muijres, F. T. (2016). The aerodynamics and control of free flight manoeuvres in *Drosophila*. *Phil. Trans. R. Soc. B* 371, 20150388. 10.1098/rstb.2015.0388 27528778 PMC4992712

[JEB251675C17] Dickerson, B. H., De Souza, A. M., Huda, A. and Dickinson, M. H. (2019). Flies regulate wing motion via active control of a dual-function gyroscope. *Curr. Biol.* 29, 3517-3524.e3. 10.1016/j.cub.2019.08.065 31607538 PMC7307274

[JEB251675C18] Dickinson, M. H., Farley, C. T., Full, R. J., Koehl, M. A. R., Kram, R. and Lehman, S. (2000). How animals move: an integrative view. *Science* 288, 100-106. 10.1126/science.288.5463.100 10753108

[JEB251675C19] Duistermars, B. J., Chow, D. M. and Frye, M. A. (2009). Flies require bilateral sensory input to track odor gradients in flight. *Curr. Biol.* 19, 1301-1307. 10.1016/j.cub.2009.06.022 19576769 PMC2726901

[JEB251675C20] Fabian, S. T., Sondhi, Y., Allen, P. E., Theobald, J. C. and Lin, H.-T. (2024). Why flying insects gather at artificial light. *Nat. Commun.* 15, 689. 10.1038/s41467-024-44785-3 38291028 PMC10827719

[JEB251675C21] Faust, R. (1952). Untersuchungen zum Halterenproblem. *Zool. Jahrb. Physiol.* 63, 325-366.

[JEB251675C22] Frederiksen, R. and Warrant, E. J. (2008). Visual sensitivity in the crepuscular owl butterfly *Caligo memnon* and the diurnal blue morpho *Morpho peleides* : a clue to explain the evolution of nocturnal apposition eyes? *J. Exp. Biol.* 211, 844-851. 10.1242/jeb.012179 18310109

[JEB251675C23] Fuller, S. B., Straw, A. D., Peek, M. Y., Murray, R. M. and Dickinson, M. H. (2014). Flying *Drosophila* stabilize their vision-based velocity controller by sensing wind with their antennae. *Proc. Natl. Acad. Sci. USA* 111, E1182-E1191. 10.1073/pnas.1323529111 24639532 PMC3977237

[JEB251675C24] Gibson, G. (1995). A behavioural test of the sensitivity of a nocturnal mosquito, *Anopheles gambiae*, to dim white, red and infra-red light. *Physiol. Entomol.* 20, 224-228. 10.1111/j.1365-3032.1995.tb00005.x

[JEB251675C25] Goulard, R., Vercher, J.-L. and Viollet, S. (2016). To crash or not to crash: how do hoverflies cope with free-fall situations and weightlessness? *J. Exp. Biol.* 219, 2497-2503. 10.1242/jeb.141150 27535987

[JEB251675C26] Goulard, R., Verbe, A., Vercher, J.-L. and Viollet, S. (2018). Role of the light source position in freely falling hoverflies’ stabilization performances. *Biol. Lett* 14, 20180051. 10.1098/rsbl.2018.0051 29794004 PMC6012696

[JEB251675C27] Green, J., Adachi, A., Shah, K. K., Hirokawa, J. D., Magani, P. S. and Maimon, G. (2017). A neural circuit architecture for angular integration in *Drosophila*. *Nature* 546, 101-106. 10.1038/nature22343 28538731 PMC6320684

[JEB251675C28] Hardie, R. C. and Juusola, M. (2015). Phototransduction in *Drosophila*. *Curr. Opin. Neurobiol.* 34, 37-45. 10.1016/j.conb.2015.01.008 25638280

[JEB251675C29] Henderson, S. R., Reuss, H. and Hardie, R. C. (2000). Single photon responses in *Drosophila* photoreceptors and their regulation by Ca^2+^. *J. Physiol.* 524, 179-194. 10.1111/j.1469-7793.2000.00179.x 10747191 PMC2269851

[JEB251675C30] Hengstenberg, R. (1988). Mechanosensory control of compensatory head roll during flight in the blowfly *Calliphora erythrocephala* Meig. *J. Comp. Physiol. A* 163, 151-165. 10.1007/BF00612425

[JEB251675C31] Hengstenberg, R. (1993). Multisensory control in insect oculomotor systems. *Rev. Oculomot. Res.* 5, 285-298. 8420553

[JEB251675C32] Honkanen, A., Takalo, J., Heimonen, K., Vähäsöyrinki, M. and Weckström, M. (2014). Cockroach optomotor responses below single photon level. *J. Exp. Biol.* 217, 4262-4268. 10.1242/jeb.112425 25472974

[JEB251675C33] Horn, E. and Lang, H.-G. (1978). Positional head reflexes and the role of the prosternal organ in the walking fly, *Calliphora erythrocephala*. *J. Comp. Physiol.* 126, 137-146. 10.1007/BF00666366

[JEB251675C34] Jakobi, T., Kolomenskiy, D., Ikeda, T., Watkins, S., Fisher, A., Liu, H. and Ravi, S. (2018). Bees with attitude: the effects of directed gusts on flight trajectories. *Biol. Open* 7, bio034074. 10.1242/bio.034074 30135080 PMC6215418

[JEB251675C35] Juusola, M. and Song, Z. (2017). How a fly photoreceptor samples light information in time. *J. Physiol.* 595, 5427-5437. 10.1113/JP273645 28233315 PMC5556158

[JEB251675C36] Kamikouchi, A., Inagaki, H. K., Effertz, T., Hendrich, O., Fiala, A., Göpfert, M. C. and Ito, K. (2009). The neural basis of *Drosophila* gravity-sensing and hearing. *Nature* 458, 165-171. 10.1038/nature07810 19279630

[JEB251675C37] Kelber, A., Warrant, E. J., Pfaff, M., Wallén, R., Theobald, J. C., Wcislo, W. T. and Raguso, R. A. (2006). Light intensity limits foraging activity in nocturnal and crepuscular bees. *Behav. Ecol.* 17, 63-72. 10.1093/beheco/arj001

[JEB251675C38] Kendzel, M. J., Parlin, A. F. and Guerra, P. A. (2023). Gravisensation and modulation of gravitactic responses by other sensory cues in the monarch butterfly (*Danaus plexippus*). *J. Exp. Biol.* 226, jeb245451. 10.1242/jeb.245451 37818736 PMC10651108

[JEB251675C39] Ketkar, M. D., Shao, S., Gjorgjieva, J. and Silies, M. (2023). Multifaceted luminance gain control beyond photoreceptors in *Drosophila*. *Curr. Biol.* 33, 2632-2645.e6. 10.1016/j.cub.2023.05.024 37285845

[JEB251675C40] Kevan, P. and Baker, H. (1983). Insects as flower visitors and pollinators. *Annu. Rev. Entomol.* 28, 407-453. 10.1146/annurev.en.28.010183.002203

[JEB251675C41] Kindermann, U. and Hertel, H. (1986). The time course of dark adaptation in the bee: a phototactic and electrophysiological investigation. *Physiol. Entomol.* 11, 23-28. 10.1111/j.1365-3032.1986.tb00387.x

[JEB251675C42] Kladt, N. and Reiser, M. B. (2023). *Drosophila* antennae are dispensable for gravity orientation. *bioRxiv*, 2023–03. 10.1101/2023.03.08.531317

[JEB251675C43] Land, M. F. and Collett, T. (1974). Chasing behaviour of houseflies (*Fannia canicularis*). *J. Comp. Physiol.* 89, 331-357. 10.1007/BF00695351

[JEB251675C44] Land, M. F., Gibson, G. and Horwood, J. (1997). Mosquito eye design: conical rhabdoms are matched to wide aperture lenses. *Proc. R. Soc. Lond. B* 264, 1183-1187. 10.1098/rspb.1997.0163

[JEB251675C45] Leonte, M.-B., Leonhardt, A., Borst, A. and Mauss, A. S. (2021). Aerial course stabilization is impaired in motion-blind flies. *J. Exp. Biol.* 224, jeb242219. 10.1242/jeb.242219 34297111

[JEB251675C46] Liu, L. and Sun, M. (2019). Dynamic flight stability of hovering mosquitoes. *J. Theor. Biol.* 464, 149-158. 10.1016/j.jtbi.2018.12.038 30597152

[JEB251675C47] Lyu, C., Abbott, L. and Maimon, G. (2022). Building an allocentric travelling direction signal via vector computation. *Nature* 601, 92-97. 10.1038/s41586-021-04067-0 34912112 PMC11104186

[JEB251675C48] Mamiya, A. and Dickinson, M. H. (2015). Antennal mechanosensory neurons mediate wing motor reflexes in flying *Drosophila*. *J. Neurosci.* 35, 7977-7991. 10.1523/JNEUROSCI.0034-15.2015 25995481 PMC6795184

[JEB251675C49] Manning, A. (1967). Antennae and sexual receptivity in *Drosophila melanogaster* females. *Science* 158, 136-137. 10.1126/science.158.3797.136 6054813

[JEB251675C50] Maya, R., Lerner, N., Ben-Dov, O., Pons, A. and Beatus, T. (2023). A hull reconstruction–reprojection method for pose estimation of free-flying fruit flies. *J. Exp. Biol.* 226, jeb245853. 10.1242/jeb.245853 37795876 PMC10629692

[JEB251675C51] Menzel, R., Ventura, D. F., Hertel, H., De Souza, J. M. and Greggers, U. (1986). Spectral sensitivity of photoreceptors in insect compound eyes: Comparison of species and methods. *J. Comp. Physiol.* 158, 165-177. 10.1007/BF01338560

[JEB251675C52] Muijres, F. T., Elzinga, M. J., Melis, J. M. and Dickinson, M. H. (2014). Flies evade looming targets by executing rapid visually directed banked turns. *Science* 344, 172-177. 10.1126/science.1248955 24723606

[JEB251675C53] Muir, L. E., Thorne, M. J. and Kay, B. H. (1992). *Aedes aegypti* (Diptera: Culicidae) vision: spectral sensitivity and other perceptual parameters of the female eye. *J. Med. Entomol.* 29, 278-281. 10.1093/jmedent/29.2.278 1495042

[JEB251675C54] Müller, P. and Robert, D. (2001). A shot in the dark: the silent quest of a free-flying phonotactic fly. *J. Exp. Biol.* 204, 1039-1052. 10.1242/jeb.204.6.1039 11222123

[JEB251675C55] Nalbach, G. (1993). The halteres of the blowfly *Calliphora*: I. Kinematics and dynamics. *J. Comp. Physiol. A* 173, 293-300. 10.1007/BF00212693

[JEB251675C56] Palavalli-Nettimi, R. and Theobald, J. C. (2020). Small eyes in dim light: Implications to spatio-temporal visual abilities in *Drosophila melanogaster*. *Vision Res.* 169, 33-40. 10.1016/j.visres.2020.02.007 32163744

[JEB251675C57] Pereira, T. D., Aldarondo, D. E., Willmore, L., Kislin, M., Wang, S. S.-H., Murthy, M. and Shaevitz, J. W. (2019). Fast animal pose estimation using deep neural networks. *Nat. Methods* 16, 117-125. 10.1038/s41592-018-0234-5 30573820 PMC6899221

[JEB251675C58] Perl, I., Maya, R., Sabag, O. and Beatus, T. (2023). Lateral instability in fruit flies is determined by wing–wing interaction and wing elevation kinematics. *Phys. Fluids* 35, 041904. 10.1063/5.0138255

[JEB251675C59] Pons, A. (2023). The self-oscillation paradox in the flight motor of *Drosophila melanogaster*. *J. R. Soc. Interface* 20, 20230421. 10.1098/rsif.2023.0421 37963559 PMC10645510

[JEB251675C60] Pons, A. and Beatus, T. (2022). Distinct forms of resonant optimality within insect indirect flight motors. *J. R. Soc. Interface* 19, 20220080. 10.1098/rsif.2022.0080 35582811 PMC9114943

[JEB251675C61] Pons, A. and Beatus, T. (2023). Band-type resonance: non-discrete energetically optimal resonant states. *Nonlinear Dyn.* 111, 1161-1192. 10.1007/s11071-022-07888-4

[JEB251675C62] Pons, A., Perl, I., Ben-Dov, O., Maya, R. and Beatus, T. (2023). Solving the thoracic inverse problem in the fruit fly. *Bioinspir. Biomim.* 18, 046002. 10.1088/1748-3190/accc23 37042474

[JEB251675C63] Pringle, J. W. S. (1948). The gyroscopic mechanism of the halteres of diptera. *R. Soc. Lond. Philos. Trans. B* 233, 347-384. 10.1098/rstb.1948.0007

[JEB251675C64] Reber, T., Vähäkainu, A., Baird, E., Weckström, M., Warrant, E. and Dacke, M. (2015). Effect of light intensity on flight control and temporal properties of photoreceptors in bumblebees. *J. Exp. Biol.* 218, 1339-1346. 10.1242/jeb.113886 25750416

[JEB251675C65] Reid, S. F., Narendra, A., Hemmi, J. M. and Zeil, J. (2011). Polarised skylight and the landmark panorama provide night-active bull ants with compass information during route following. *J. Exp. Biol.* 214, 363-370. 10.1242/jeb.049338 21228195

[JEB251675C66] Ristroph, L., Bergou, A. J., Ristroph, G., Coumes, K., Berman, G. J., Guckenheimer, J., Wang, Z. J. and Cohen, I. (2010). Discovering the flight autostabilizer of fruit flies by inducing aerial stumbles. *Proc. Natl Acad. Sci. USA* 107, 4820-4824. 10.1073/pnas.1000615107 20194789 PMC2841947

[JEB251675C67] Sane, S. P. (2003). The aerodynamics of insect flight. *J. Exp. Biol.* 206, 4191-4208. 10.1242/jeb.00663 14581590

[JEB251675C68] Sharkey, C. R., Blanco, J., Leibowitz, M. M., Pinto-Benito, D. and Wardill, T. J. (2020). The spectral sensitivity of Drosophila photoreceptors. *Sci. Rep.* 10, 18242. 10.1038/s41598-020-74742-1 33106518 PMC7588446

[JEB251675C69] Souman, J. L., Frissen, I., Sreenivasa, M. N. and Ernst, M. O. (2009). Walking straight into circles. *Curr. Biol.* 19, 1538-1542. 10.1016/j.cub.2009.07.053 19699093

[JEB251675C70] Spiewok, S. and Schmolz, E. (2006). Changes in temperature and light alter the flight speed of hornets (*Vespa crabro* L.). *Physiol. Biochem. Zool.* 79, 188-193. 10.1086/498181 16380940

[JEB251675C71] Sponberg, S., Dyhr, J. P., Hall, R. W. and Daniel, T. L. (2015). Luminance-dependent visual processing enables moth flight in low light. *Science* 348, 1245-1248. 10.1126/science.aaa3042 26068850

[JEB251675C72] Stein, B. (1993). *The Merging of the Senses*. MIT Press.

[JEB251675C73] Stewart, F. J., Baker, D. A. and Webb, B. (2010). A model of visual–olfactory integration for odour localisation in free-flying fruit flies. *J. Exp. Biol.* 213, 1886-1900. 10.1242/jeb.026526 20472776

[JEB251675C74] Sun, M. (2014). Insect flight dynamics: stability and control. *Rev. Mod. Phys.* 86, 615. 10.1103/RevModPhys.86.615

[JEB251675C75] Suver, M. P., Matheson, A. M. M., Sarkar, S., Damiata, M., Schoppik, D. and Nagel, K. I. (2019). Encoding of wind direction by central neurons in *Drosophila*. *Neuron* 102, 828-842. 10.1016/j.neuron.2019.03.012 30948249 PMC6533146

[JEB251675C76] Taylor, G. K. and Krapp, H. G. (2007). Sensory systems and flight stability: what do insects measure and why? *Adv. Insect Physiol.* 34, 231-316. 10.1016/S0065-2806(07)34005-8

[JEB251675C77] Theobald, J. C., Coates, M. M., Wcislo, W. T. and Warrant, E. J. (2007). Flight performance in night-flying sweat bees suffers at low light levels. *J. Exp. Biol.* 210, 4034-4042. 10.1242/jeb.003756 17981871

[JEB251675C78] Theriault, D. H., Fuller, N. W., Jackson, B. E., Bluhm, E., Evangelista, D., Wu, Z., Betke, M. and Hedrick, T. L. (2014). A protocol and calibration method for accurate multi-camera field videography. *J. Exp. Biol.* 217, 1843-1848. 10.1242/jeb.100529 24577444

[JEB251675C79] Van Breugel, F. and Dickinson, M. H. (2012). The visual control of landing and obstacle avoidance in the fruit fly *Drosophila melanogaster*. *J. Exp. Biol.* 215, 1783-1798. 10.1242/jeb.066498 22573757

[JEB251675C80] van Breugel, F., Riffell, J., Fairhall, A. and Dickinson, M. H. (2015). Mosquitoes use vision to associate odor plumes with thermal targets. *Curr. Biol.* 25, 2123-2129. 10.1016/j.cub.2015.06.046 26190071 PMC4546539

[JEB251675C81] Vance, J., Faruque, I. and Humbert, J. (2013). Kinematic strategies for mitigating gust perturbations in insects. *Bioinspir. Biomim.* 8, 16004. 10.1088/1748-3182/8/1/016004 23302326

[JEB251675C82] Vance, J. T., Altshuler, D. L., Dickson, W. B., Dickinson, M. H. and Roberts, S. P. (2014). Hovering flight in the honeybee *Apis mellifera*: kinematic mechanisms for varying aerodynamic forces. *Physiol. Biochem. Zool.* 87, 870-881. 10.1086/678955 25461650

[JEB251675C83] Verbe, A., Martinez, D. and Viollet, S. (2023). Sensory fusion in the hoverfly righting reflex. *Sci. Rep.* 13, 6138. 10.1038/s41598-023-33302-z 37061548 PMC10105705

[JEB251675C84] Vinauger, C., Van Breugel, F., Locke, L. T., Tobin, K. K. S., Dickinson, M. H., Fairhall, A. L., Akbari, O. S. and Riffell, J. A. (2019). Visual-olfactory integration in the human disease vector mosquito *Aedes aegypti*. *Curr. Biol.* 29, 2509-2516.e5. 10.1016/j.cub.2019.06.043 31327719 PMC6771019

[JEB251675C85] Vogel, S. (1966). Flight in *Drosophila* I. Flight performance of tethered flies. *J. Exp. Biol.* 44, 567-578. 10.1242/jeb.44.3.567

[JEB251675C86] Von Schilcher, F. (1976). The role of auditory stimuli in the courtship of *Drosophila melanogaster*. *Anim. Behav.* 24, 18-26. 10.1016/S0003-3472(76)80095-4

[JEB251675C87] Walthall, W. W. and Hartman, H. B. (1981). Receptors and giant interneurons signaling gravity orientation information in the cockroach *Arenivaga*. *J. Comp. Physiol.* 142, 359-369. 10.1007/BF00605448

[JEB251675C88] Warrant, E. J. (2015). Visual tracking in the dead of night. *Science* 348, 1212-1213. 10.1126/science.aab2185 26068837

[JEB251675C89] Warrant, E. J. (2017). The remarkable visual capacities of nocturnal insects: vision at the limits with small eyes and tiny brains. *Phil. Trans. R. Soc. B* 372, 20160063. 10.1098/rstb.2016.0063 28193808 PMC5312013

[JEB251675C90] Warrant, E. J. (2008). Seeing in the dark: vision and visual behaviour in nocturnal bees and wasps. *J. Exp. Biol.* 211, 1737-1746. 10.1242/jeb.015396 18490389

[JEB251675C91] Warrant, E. J., Kelber, A., Gislén, A., Greiner, B., Ribi, W. and Wcislo, W. T. (2004). Nocturnal vision and landmark orientation in a tropical halictid bee. *Curr. Biol.* 14, 1309-1318. 10.1016/j.cub.2004.07.057 15296747

[JEB251675C92] Wenner, A. (1963). The flight speed of honeybees: a quantitative approach. *J. Apic. Res.* 2, 25-32. 10.1080/00218839.1963.11100053

[JEB251675C93] Wessnitzer, J. and Webb, B. (2006). Multimodal sensory integration in insects—towards insect brain control architectures. *Bioinspir. Biomim* 1, 63. 10.1088/1748-3182/1/3/001 17671308

[JEB251675C94] Whitehead, S. C., Beatus, T., Canale, L. and Cohen, I. (2015). Pitch perfect: how fruit flies control their body pitch angle. *J. Exp. Biol.* 218, 3508-3519. 10.1242/jeb.122622 26385332

[JEB251675C95] Whitehead, S. C., Leone, S., Lindsay, T., Meiselman, M. R., Cowan, N. J., Dickinson, M. H., Yapici, N., Stern, D. L., Shirangi, T. and Cohen, I. (2022). Neuromuscular embodiment of feedback control elements in *Drosophila* flight. *Sci. Adv.* 8, eabo7461. 10.1126/sciadv.abo7461 36516241 PMC9750141

[JEB251675C96] Wolf, E. and Zerrahn-Wolf, G. (1935). The dark adaptation of the eye of the honey bee. *J. Gen. Physiol.* 19, 229-237. 10.1085/jgp.19.2.229 19872922 PMC2141435

[JEB251675C97] Xu, N. and Sun, M. (2013). Lateral dynamic flight stability of a model bumblebee in hovering and forward flight. *J. Theor. Biol.* 319, 102-115. 10.1016/j.jtbi.2012.11.033 23220347

[JEB251675C98] Yamaguchi, S., Desplan, C. and Heisenberg, M. (2010). Contribution of photoreceptor subtypes to spectral wavelength preference in *Drosophila*. *Proc. Natl. Acad. Sci. USA* 107, 5634-5639. 10.1073/pnas.0809398107 20212139 PMC2851746

[JEB251675C99] Yarger, A. M. and Fox, J. L. (2016). Dipteran halteres: perspectives on function and integration for a unique sensory organ. *Integr. Comp. Biol.* 56, 865-876. 10.1093/icb/icw086 27413092

