# Supplementary material for: Flight in the dark: different responses to darkness in flying insects
Source: J Exp Biol. 2026 May 20;229(10):jeb251675. doi: 10.1242/jeb.251675 (PMC13245909; doi:10.1242/jeb.251675)
Supplement: Supplementary information [file jexbio-229-251675-s1.pdf]

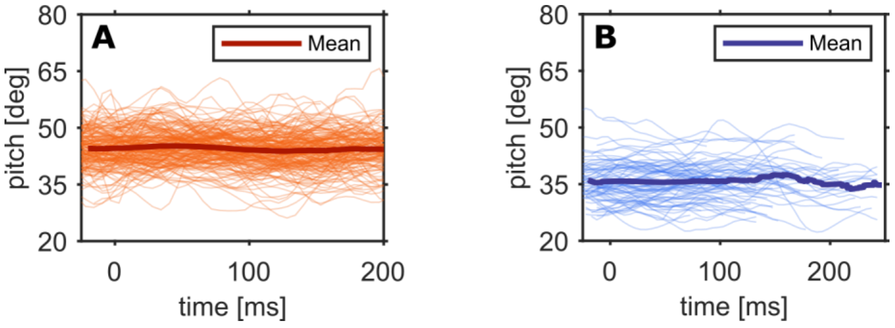

Fig. S1. Body pitch kinematics in unperturbed experiments show no maneuver similar to the dark response. Plots show the body pitch during control experiments, in which the fruit flies (A) and mosquitoes (B) were flying freely in constant lit conditions. Data includes 192 fruit fly trajectories and 82 mosquito trajectories.

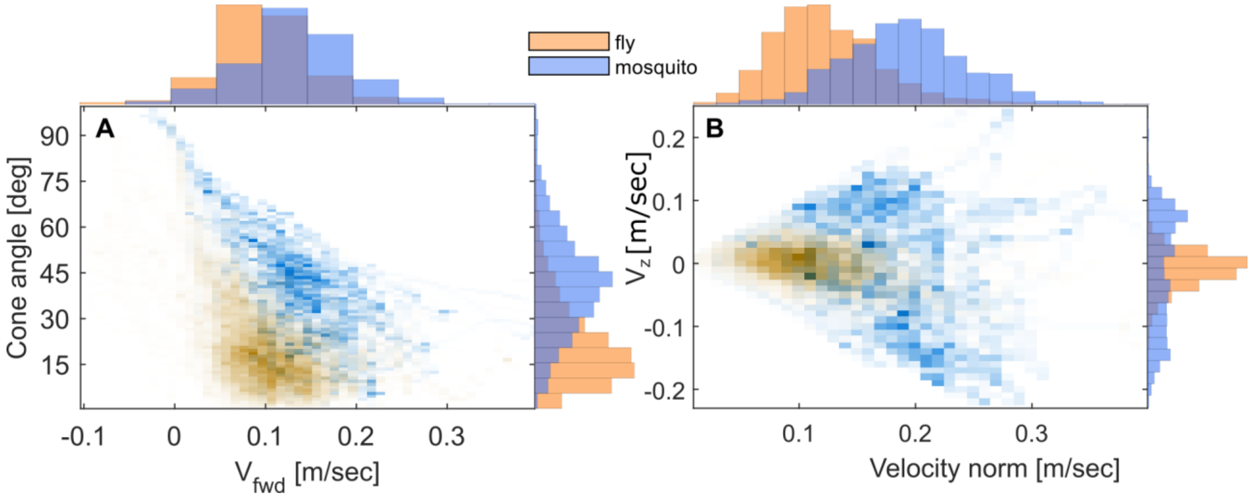

Fig. S2. Nominal velocity distributions. 2D histograms represented by color intensity, along with marginal histogram for separate axes. **(A)** Cone angle *vs.* V_{fwd}. **(B)** V_Z *vs.* the norm of velocity vector.

Journal of Experimental Biology • Supplementary information

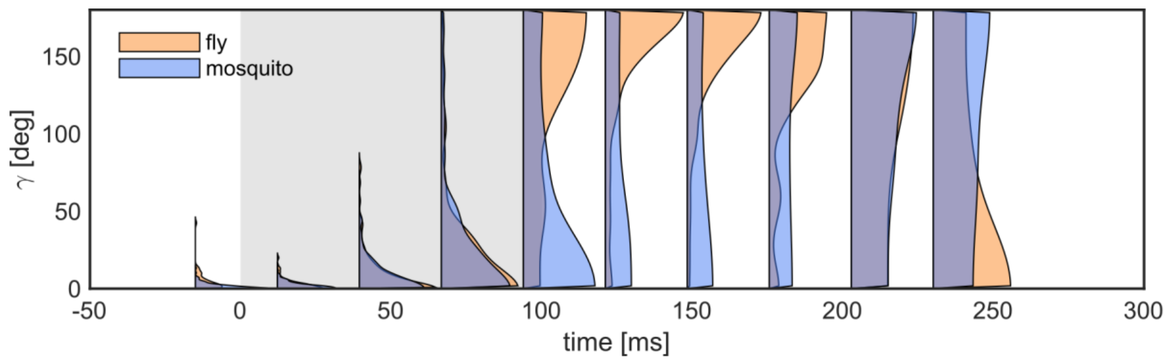

Fig. S3. Relative flight direction in 100 ms pulse perturbations. The distributions of γ – the change of horizontal flight direction – under a dark pulse perturbation of 100 ms (grayed area) for 57 fly events and 119 mosquito events. The distributions are shown at distinct times.

Journal of Experimental Biology • Supplementary information

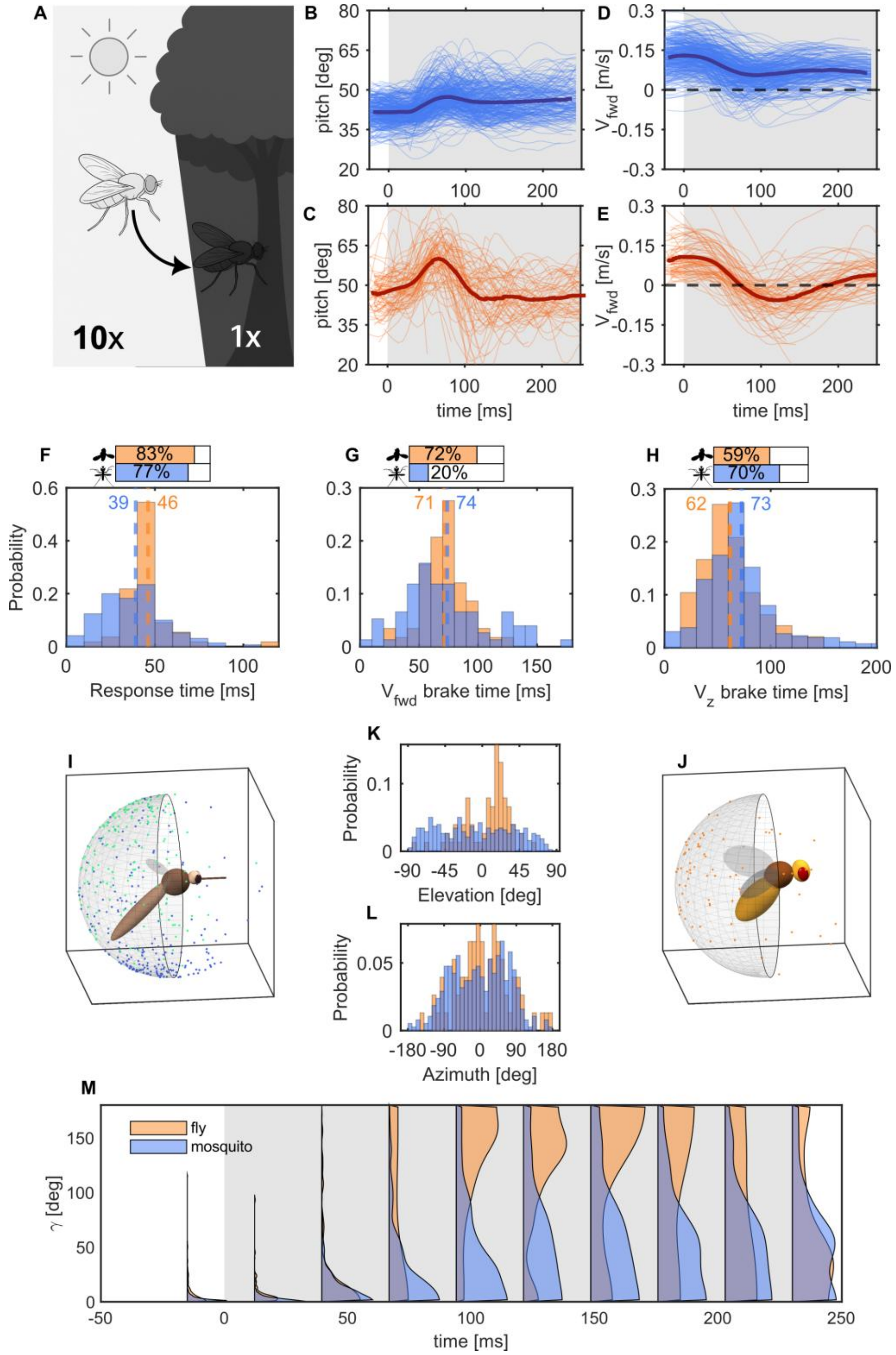

Fig. S4. The response of fruit flies and mosquitoes to a partial darkness step perturbation is similar to their response to a complete darkness step perturbation. (A) Under a partial darkness step perturbation, light intensity reduced 10-fold. The illustration shows a possible scenario for such a perturbation, where an insect flies from a sunlit to a shaded area under tree canopy. (B-E) Flight kinematics for partial darkness step perturbation, with means shown in bold. Body pitch angle for fruit flies (B) and mosquitoes (C) across all measured events. Body forward velocity for fruit flies (D) and mosquitoes (E). (F-H) Distributions of response times for fruit flies and mosquitoes under partial darkness step perturbations: (F) acceleration-based response times; (G) V_{fwd} brake times; (H) V_z brake times. (I-L) The direction of maximum acceleration during the dark response maneuver, calculated in the insects' body frame of reference. Spherical scatter plots of the distribution of direction for fruit flies (I) and mosquitoes (J); Azimuth (K) and elevation angles (L) distribution of maximum acceleration direction for both species. (M) Distributions of γ (the change of horizontal flight direction) in all partial darkness step perturbations for fruit flies and mosquitoes in distinct times.

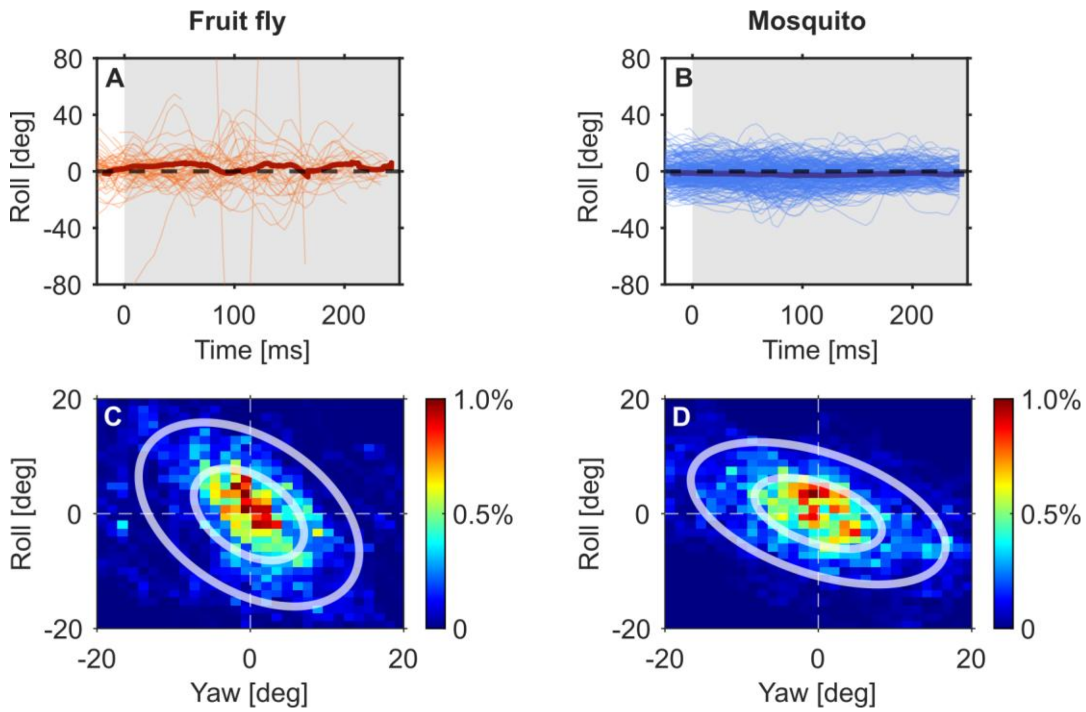

Fig. S5. The roll angle kinematics during the dark-response maneuvers is coupled to yaw. (A,B) The roll angle vs. time for the entire dataset of fruit flies **(A)** and mosquitoes **(B)** under darkness step perturbation. (C,D) a two dimensional histogram of the roll and yaw angles for the same fruit fly (C) and mosquito (D) datasets. The ellipses represent Gaussian fits to each histogram. The correlation coefficient between the roll and yaw angles are -0.41 for the fruit flies and -0.46 for the mosquitoes. The p-values for these correlation coefficients are $p \ll 10^{-6}$.

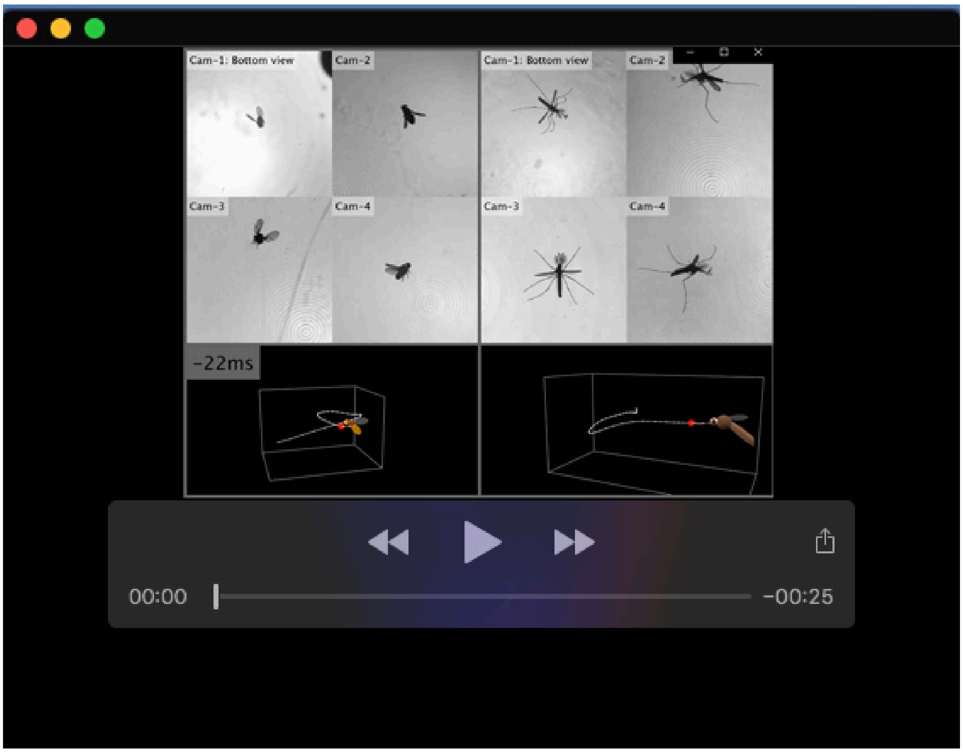

Movie 1. Representative responses of a fruit fly and a mosquito to a darkness *step perturbation*, where at t=0 light was reduced 500,000 fold from 34 lux to $6.8{\cdot}10^{-5}$ lux. The darkness is illustrated by intensity inversion of each camera view. The insect center-of-mass trajectories are plotted. Dots on the trajectory indicate the perturbation onset time.

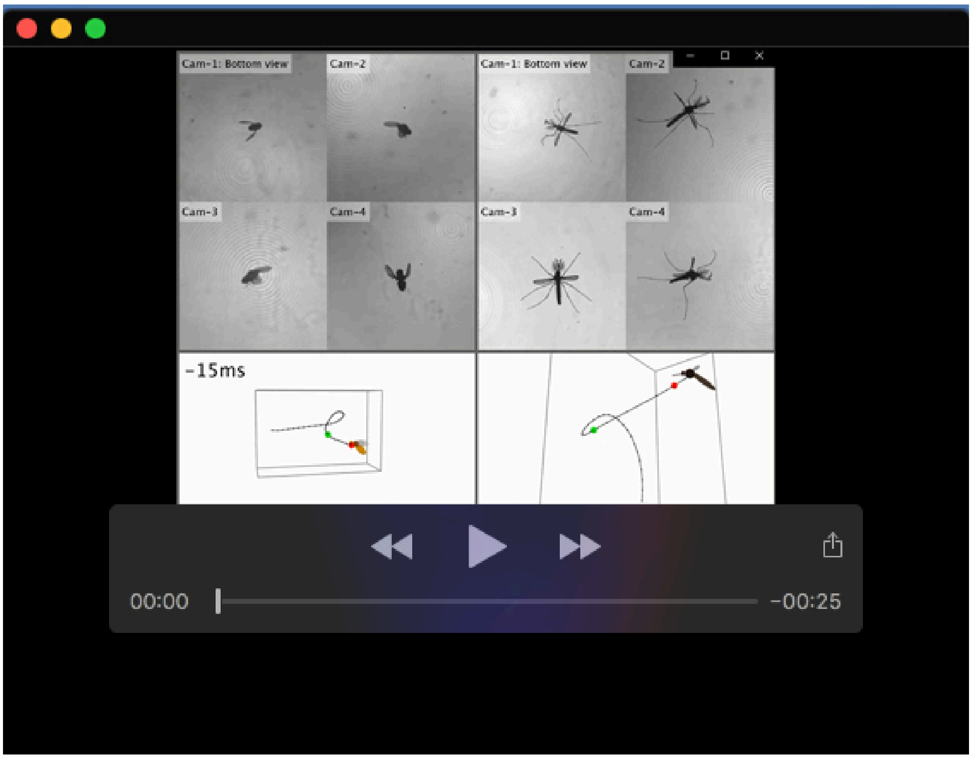

Movie 2. Representative responses of a fruit fly and a mosquito to a darkness 60ms pulse perturbation starting at $t = 0$, when light was reduced 500,000 fold from 34 lux to $6.8{\cdot}10^{-5}$ lux. The darkness is illustrated by intensity inversion of each camera view. The insect center-of-mass trajectories are plotted. Dots on the trajectory indicate the perturbation onset time.

Journal of Experimental Biology • Supplementary information

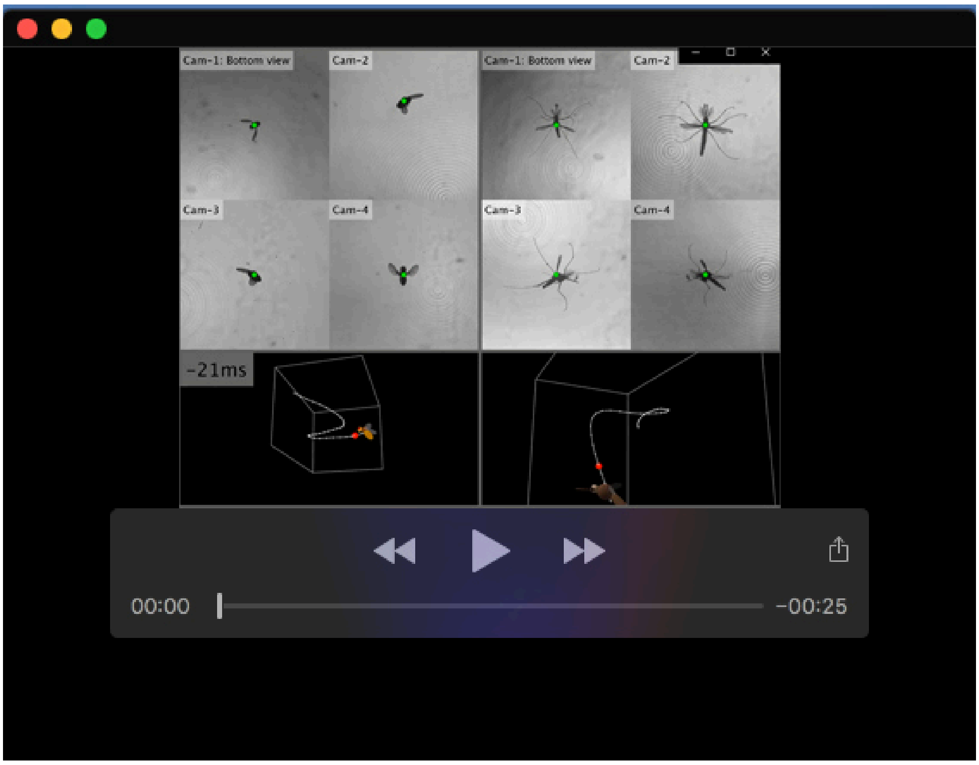

Movie 3. Representative responses of a fruit fly and a mosquito to a partial darkness step perturbation started at $t = 0$. Light was reduced 10 fold from 34 lux

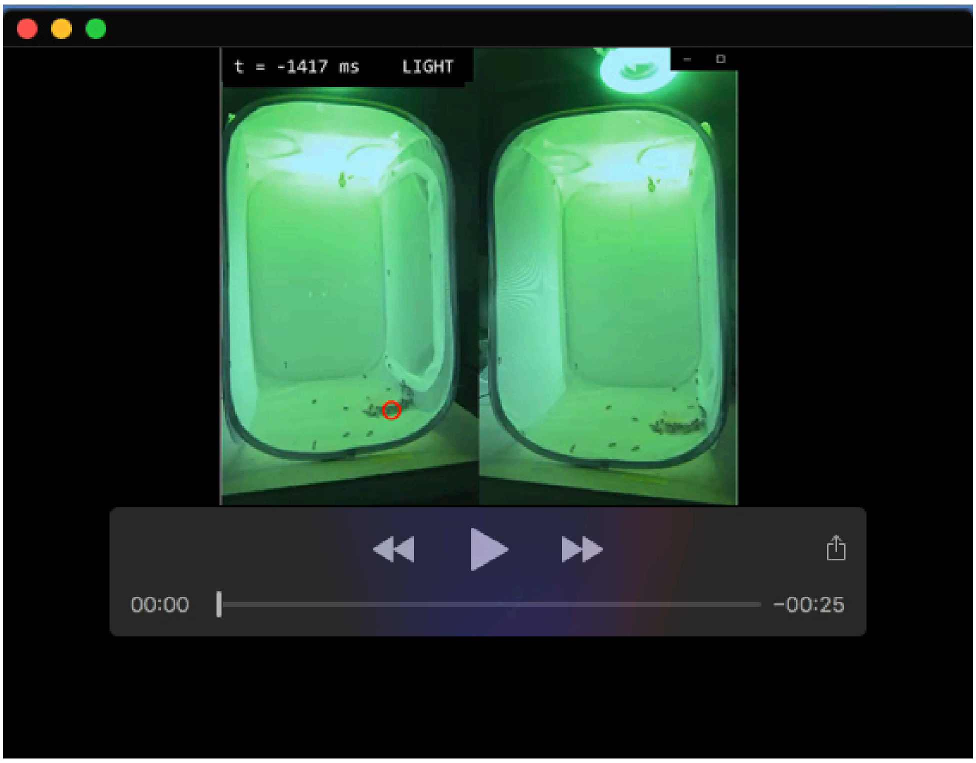

Movie 4. Bee response to sudden darkness step perturbation at t=0 from 2,600 lux to $9.6 \cdot 10^{-4}$ lux (2,700,000 fold reduction). The trajectories of two bees are marked in red and yellow, as described in Figure 2.

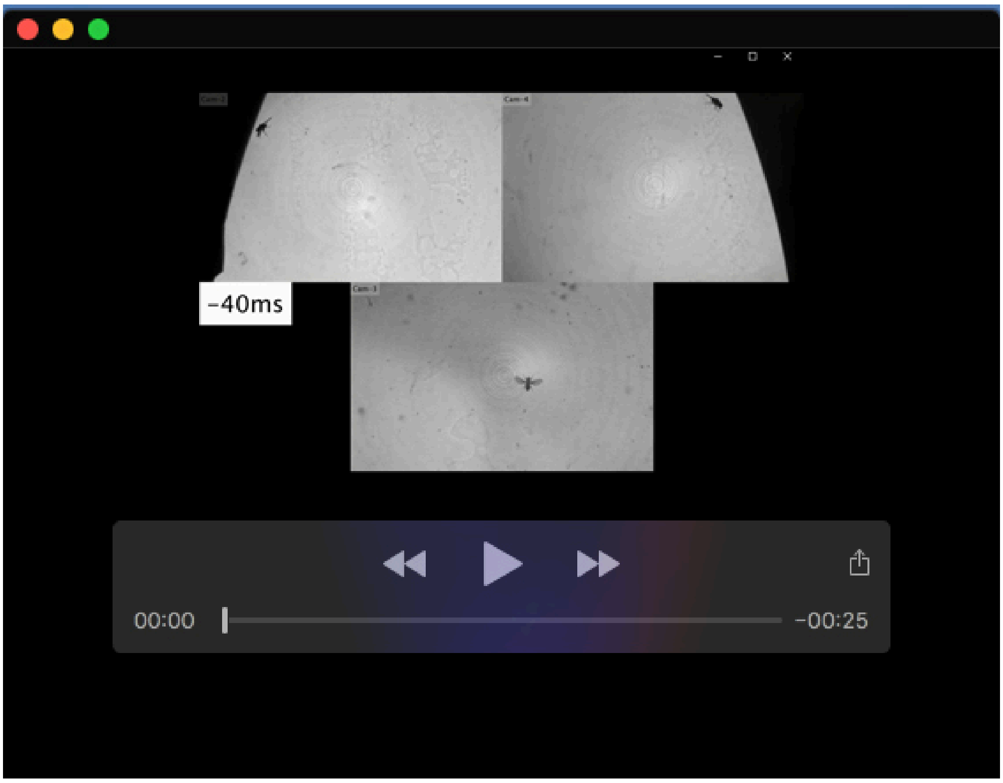

Movie 5. A fruit fly performing free flight under continuous very low-light of $6.8{\cdot}10^{-5}$ lux. The fly took off volitionally in complete darkness. To get to the recorded volume located in the middle of the flight chamber the fly had to fly in a controlled and stable manner.

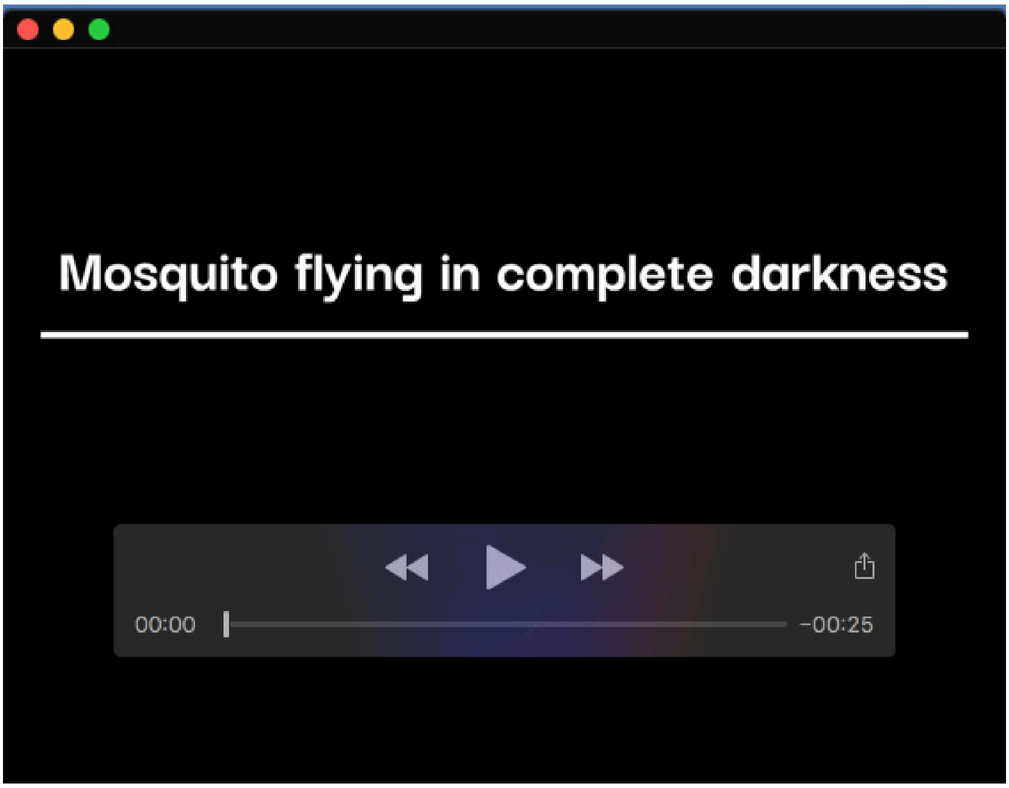

Movie 6. A mosquito performing free flight in under continuous very low-light of $6.8{\cdot}10^{-5}$ lux. The mosquito took off volitionally in complete darkness. To get to the recorded volume located in the middle of the flight chamber the mosquito had to fly in a controlled and stable manner.

Journal of Experimental Biology • Supplementary information

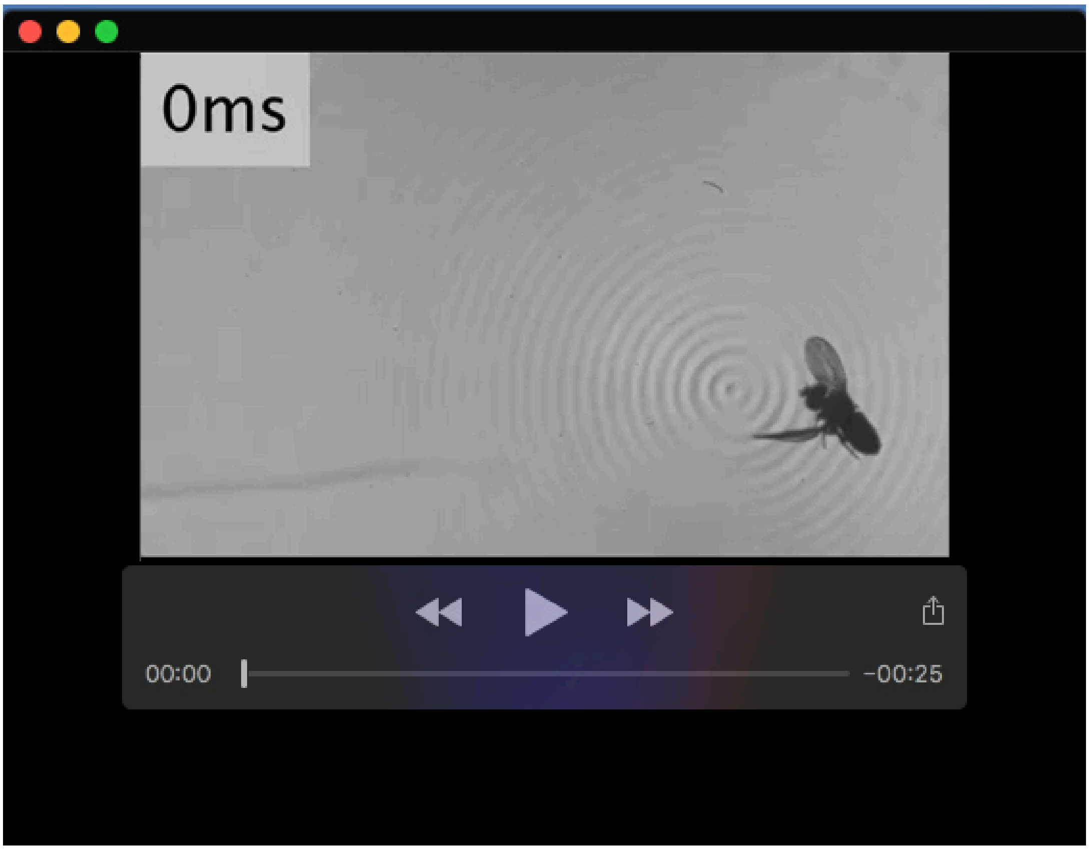

Movie 7. Leg extension response of a fruit fly following a darkness step perturbation that started at $t = 0$, from 34 lux to $6.8 \cdot 10^{-5}$ lux (500,000 fold reduction). The movie is paused when the onset of leg extension is clearly visible.
